# Gastric cancer treatment: recent progress and future perspectives

**DOI:** 10.1186/s13045-023-01451-3

**Published:** 2023-05-27

**Authors:** Wen-Long Guan, Ye He, Rui-Hua Xu

**Affiliations:** 1 Department of Medical Oncology, State Key Laboratory of Oncology in South China, Collaborative Innovation Center for Cancer Medicine, Sun Yat-Sen University Cancer Center, 651 Dongfeng Road East, Guangzhou, 510060 People’s Republic of China; 2 Research Unit of Precision Diagnosis and Treatment for Gastrointestinal Cancer, Chinese Academy of Medical Sciences, Guangzhou, 510060 People’s Republic of China

**Keywords:** Gastric cancer, Perioperative chemotherapy, Immunotherapy, Targeted therapy

## Abstract

Gastric cancer (GC) is one of the most common malignancies worldwide. Most patients are diagnosed at advanced stages due to the subtle symptoms of earlier disease and the low rate of regular screening. Systemic therapies for GC, including chemotherapy, targeted therapy and immunotherapy, have evolved significantly in the past few years. For resectable GC, perioperative chemotherapy has become the standard treatment. Ongoing investigations are exploring the potential benefits of targeted therapy or immunotherapy in the perioperative or adjuvant setting. For metastatic disease, there have been notable advancements in immunotherapy and biomarker-directed therapies recently. Classification based on molecular biomarkers, such as programmed cell death ligand 1 (PD-L1), microsatellite instability (MSI), and human epidermal growth factor receptor 2 (HER2), provides an opportunity to differentiate patients who may benefit from immunotherapy or targeted therapy. Molecular diagnostic techniques have facilitated the characterization of GC genetic profiles and the identification of new potential molecular targets. This review systematically summarizes the main research progress in systemic treatment for GC, discusses current individualized strategies and presents future perspectives.

## Background

Gastric cancer (GC) is the fifth most common malignant tumor and the fourth leading cause of cancer-associated death worldwide [1, 2]. The incidence varies geographically across the globe, with the highest incidence in Eastern Asia (Japan and Mongolia) and Eastern Europe, whereas incidence rates in Northern Europe and Northern America are generally low, comparable to African regions [2]. Notably, the incidence of gastric cancer among young adults (aged < 50 years) in recent years has been progressively rising in both low-risk and high-risk countries. Aside from *Helicobacter Pylori* infection, the occurrence of GC has been linked to genetic risk factors as well as lifestyle factors, such as alcohol consumption and smoking [3–6].

Despite the high incidence of GC, most patients are unfortunately diagnosed at advanced stages with dismal prognoses due to the lack of distinguishing clinical indications [7, 8]. Systemic chemotherapy is the mainstay treatment for metastatic GC (mGC), with a median overall survival (OS) of ~ 12 months for patients treated with conventional chemotherapy [9]. Intratumoral and intertumoral heterogeneity are the prominent features of GC that partly contribute to its poor prognosis. However, histological classifications alone are insufficient to effectively stratify patients for individualized treatment and improve patients’ clinical outcomes [10]. Therefore, cutting-edge diagnostic techniques and drugs are of fundamental importance for better characterizing molecular profiles and identifying potential novel therapeutic targets for GC patients [11–13].

Trastuzumab, a monoclonal antibody targeting Human Epidermal Receptor 2 (HER2), was the first approved targeted therapy for GC. However, after the ToGA study, progress in the development of treatments for gastric cancer stalled for nearly a decade [14]. Emerging advances in immunotherapy, particularly in anti-HER2 therapy, and various biomarker-directed therapies in GC have recently broken this trend. For example, anti-programmed cell death 1 (PD-1) antibodies have demonstrated impressive efficacy and prolonged survival in untreated MSI-H/dMMR mGC patients [15]. Substantial breakthroughs in the treatment of gastric cancer have been achieved with novel anti-HER2 therapeutic agents, such as T-DXd and disitamab vedotin (RC48) [16]. In addition, in light of the success of immunotherapy and targeted therapy as first-line treatments for advanced gastric cancer, ongoing research is investigating their potential to advance the treatment of patients with locally advanced stage GC.

**Fig. 1:**
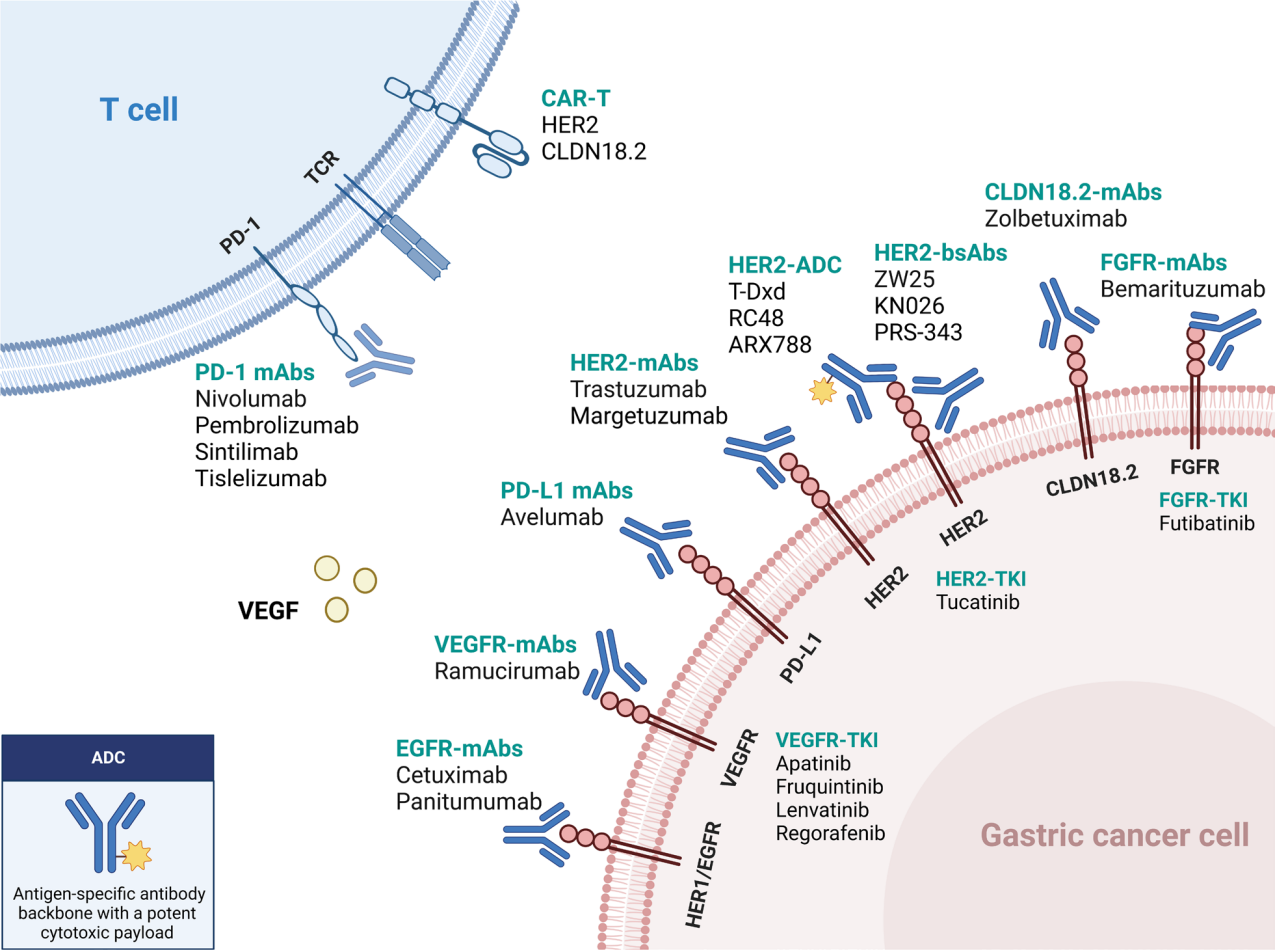
Updated immunotherapy and targeted therapy for gastric cancer. This algorithm provides guidance for selecting currently available immunotherapy and targeted therapy based on different biomarkers

The treatment landscape of gastric cancer has evolved significantly in the past few years, with the emergence of new immunotherapy and targeted therapies for patients at various stages of the disease (Fig. 1). In this review, we systematically summarize the pivotal clinical trials in GC treatment and provide an update on the management of localized and metastatic gastric cancer. We also discuss the developments in immunotherapy and targeted therapy and highlight current individualized treatments and future perspectives.

## Management for localized GC

Radical surgery is the primary treatment for resectable gastric cancer. Several therapeutic approaches have been established to lower the risk of recurrence and improve long-term survival, including perioperative chemotherapy, adjuvant chemotherapy, and adjuvant chemoradiotherapy (Table 1). They are listed as the recommended treatments for resectable localized GC in current guidelines[5, 17, 18]. Further, the addition of targeted therapy and/or immune checkpoint inhibitors (ICIs) is currently being studied in the neoadjuvant/adjuvant setting.

**Table 1:** Summary of the key phase III clinical trials of perioperative and adjuvant chemotherapy in resectable GC

Therapy line		Trial	Stage	Experimental arm	Control arm	Primary endpoints	Result (Experimental vs. Control)	References
Perioperative chemotherapy		MAGIC	Stage II or higher	3 × ECF → Surgery → 3 × ECF	Surgery	OS	PFS: HR 0.66; 95% CI 0.53–0.81; P < 0.001; 5-year OS: 36% vs. 23% (HR 0.75; 95% CI 0.60–0.93; * P* = 0.009)	[19]
		FNCLCC & FFCD	Resectable	2–3 × CF → Surgery → 3–4 × CF	Surgery	OS	5-year DFS: 34% v 19% (HR, 0.65; 95% CI 0.48 -0.89; * P* = 0.003) 5-year OS: 38% v 24% (HR, 0.69; 95% CI 0.50 -0.95; * P* = 0.02)	[20]
		FLOT4-AIO	cT2 or higher or N +	4 × FLOT → Surgery → 4 × FLOT	3 × ECF/ECX → Surgery → 3 × ECF/ECX	OS	PFS: 30 vs. 18 months (HR 0.75; 95% CI 0.62–0.91; * P* = 0.0036); OS: 50 vs. 35 months (HR 0.77; 95% CI 0.63–0.94; * P* = 0.012)	[21]
		PRODIGY	cT2-3N + , or cT4Nany	3 × DOS → Surgery → 8 × S-1	Surgery → S-1	PFS	3-year PFS: 66.3% vs. 60.2% (HR 0.70; 95% CI, 0.52–0.95; * P* = 0.023); 3-year OS: 74.2% vs. 73.4% (HR 0.84; 95% CI, 0.60–1.19; * P* = 0.338)	[22]
		RESOLVE	cT4aN + or cT4bNany	Arm C: 3 × SOX → Surgery → 5 × SOX → 3 × S-1	Arm A: Surgery → 8 × CAPOX	DFS	3-year DFS: 59.4% vs. 51.1% (HR 0.77; 95% CI, 0.61–0.97; * P* = 0.028)	[24]
Adjuvant chemotherapy		ACTS-GC	Stage II or III	Surgery → S-1 for 1 year	Surgery	OS	5-year OS: 71.7% vs. 61.1% (HR 0.669; 95% CI, 0.540 to 0.828)	[27]
		CLASSIC	Stage II-IIIB	Surgery → 8 × CAPOX	Surgery	DFS	5-year DFS: 68% vs. 53% (HR 0.58; 95% CI, 0.47 to 0.72; P < 0.0001) 5-year OS: 78% vs. 69% (HR 0.66; 95% CI, 0.51 to 0.85; * P* = 0.0015)	[25] [26]
		JACCRO GC-07	Stage III	Surgery → 1 × S-1 → 7 × Docetaxel plus S-1 → S-1 for 1 year	Surgery → S-1 for 1 year	RFS	3-year RFS: 66% vs. 50% (HR 0.632; 99.99% CI, 0.400 to 0.998; P < 0.001)	[23]
		RESOLVE	cT4aN + or cT4bNany	Arm B: Surgery → 8 × SOX	Arm A: Surgery → 8 × CAPOX	DFS	3-year DFS: 56.5% vs. 51.1% (HR 0.86; 95% CI, 0.68 to 1.07; * P* = 0.17)	[24]

### Perioperative chemotherapy

Perioperative chemotherapy has become the standard treatment for resectable localized GC. Several clinical trials have demonstrated that perioperative chemotherapy could improve the prognosis of patients with resectable GC compared to surgery alone.

The MAGIC trial marked a significant advancement in the field of perioperative chemotherapy for resectable GC. In this phase 3 study, 503 patients were enrolled with resectable gastric, gastroesophageal junction (GEJ), or lower esophageal adenocarcinoma. Patients in the experimental group received three preoperative and three postoperative cycles of epirubicin, cisplatin, and fluorouracil (ECF) [19]. The results showed that the perioperative ECF regimen could decrease tumor stage and significantly improve progression-free survival (PFS, HR 0.66; 95% CI 0.53–0.81, P < 0.001) and overall survival (OS, HR 0.75; 95% CI 0.60–0.93, *P* = 0.009). Another phase III trial conducted in 28 French centers compared radical surgery with or without perioperative cisplatin and fluorouracil (CF) chemotherapy and showed that perioperative chemotherapy led to a higher 5-year overall survival rate versus surgery alone (38% versus 24%, respectively; HR 0.69; 95% CI 0.50–0.95, *P* = 0.02) [20]. Recently, the randomized phase II/III FLOT4-AIO study compared perioperative FLOT regimen (fluorouracil, leucovorin, oxaliplatin, and docetaxel) with previous standard ECF/ECX (epirubicin, cisplatin, and fluorouracil/capecitabine) regimen in gastric or GEJ cancer patients who had cT2 or higher and nodal positive (cN +) disease [21]. The results suggested that the FLOT regimen could improve overall survival (50 months versus 35 months), confirming the role of the FLOT regimen as the new standard perioperative treatment for resectable gastric cancer [5, 18].

Since most of the clinical trials mentioned above were conducted in western countries, these perioperative regimens (ECF, CF, and FLOT) are less frequently used in Asia. In the phase III PRODIGY trial, 530 Korean patients with cT2-3N + or cT4N_any_ gastric or GEJ cancer were randomly randomized to the neoadjuvant or adjuvant group. Patients in the neoadjuvant arm underwent preoperative DOS (docetaxel, oxaliplatin, and S-1) followed by surgery and S-1 adjuvant chemotherapy, while those in the adjuvant arm received upfront radical surgery followed by S-1 chemotherapy [22]. The perioperative chemotherapy group had significantly higher rates of R0 resection and pathological complete response (pCR) (95% and 10.4%, respectively). Moreover, PFS was improved in the perioperative arm compared to the adjuvant arm (HR 0.70; 95% CI 0.52–0.95; *P* = 0.023). The major criticism of this study was that the adjuvant S-1 monotherapy was insufficient for stage III patients, considering another phase III study had demonstrated the superiority of docetaxel plus S-1 to S-1 for 3-year relapse-free survival (RFS) in stage III gastric cancer [23]. Recently, the phase III RESOLVE trial conducted in China investigated the role of perioperative S-1 plus oxaliplatin (SOX) chemotherapy versus upfront surgery followed by adjuvant chemotherapy [24]. This study recruited over 1,000 patients with cT4aN + or cT4bN_any_ gastric or GEJ adenocarcinoma, of whom over 60% had gastric cancer. Patients in the intervention group received perioperative SOX (three preoperative cycles and five postoperative cycles followed by three cycles of S-1 monotherapy). The two adjuvant groups received surgery followed by SOX or CAPOX (capecitabine and oxaliplatin) chemotherapy. These results suggested that the perioperative SOX chemotherapy could improve the 3-year disease-free survival (DFS) rate compared to adjuvant CAPOX therapy (59.4% vs. 51.1%, respectively, *P* = 0.028).

Based on the evidence shown above, perioperative chemotherapy has become the standard treatment in many countries. The FLOT regimen is the most commonly used in Western countries according to the evidence from the FLOT4-AIO study[21], while the SOX regimen is more recommended in China, based on the results of the RESOLVE study[24]. However, perioperative chemotherapy is less recommended in Japan, since evidence of the superiority of neoadjuvant chemotherapy is still lacking among Japanese patients[25].

### Adjuvant chemotherapy

Adjuvant chemotherapy is recommended for patients who undergo primary surgery and have stage II or stage III disease due to improvement in survival demonstrated by several clinical trials, particularly in Asian patients. The multi-center phase III CLASSIC trial undertaken in South Korea, China, and Taiwan compared upfront D2 surgery followed by CAPOX adjuvant chemotherapy versus D2 gastrectomy alone in patients with stage II-IIIB gastric cancer [26, 27]. Adjuvant CAPOX chemotherapy significantly improved both 5-year DFS (68% vs. 53%; HR 0.58; 95% CI, 0.47 to 0.72; P < 0.0001) and OS (78% vs. 69%; HR 0.66; 95% CI, 0.51 to 0.85; *P* = 0.0015) compared with surgery alone. Another similar phase III ACTS-GC trial from Japan randomly assigned 1,059 stage II or III GC patients to undergo D2 surgery followed by S-1 monotherapy or D2 surgery alone and showed that adjuvant S-1 monotherapy for one year led to a better 3-year OS than surgery alone (80.1% vs. 70.1%; HR 0.68; 95% CI, 0.52 to 0.87; *P* = 0.003). The survival benefit persisted after five years of follow-up [28]. Moreover, the phase III JACCRO GC-07 trial investigated the superiority of adjuvant docetaxel plus S-1 over S-1 monotherapy for pathological stage III gastric cancer [23]. The addition of docetaxel to S-1 after surgery showed a better 3-year RFS (66% vs. 50%; HR 0.632; 99.99% CI, 0.400 to 0.998; *P* < 0.001) in the second interim analysis, and the study was terminated as recommended by the independent data and safety monitoring committee. The RESOLVE trial also investigated the non-inferiority of adjuvant SOX chemotherapy compared with the CAPOX regimen. The 3-year DFS was statistically comparable between the two groups (56.5% vs. 51.1%; HR 0.86; 95% CI, 0.68 to 1.07; *P* = 0.17) [24]. Based on the results of the phase III trials presented above, several cytotoxic regimens could be used as adjuvant treatments for stage II-III GC after radical surgery, including S-1, CAPOX, SOX, and DS. The choice of regimens depends on many factors, including the pathological staging, patient performance status, and toxicity profile. In general, S-1 monotherapy is more recommended for stage II disease or for patients with poor performance status. Combination therapies such as CAPOX, SOX, or DS are often recommended for pathological stage III disease[17, 25].

GC with microsatellite instability-high (MSI-H) or mismatch-repair deficiency (dMMR) is a distinct subtype [11]. Recently, an individual-patient-data meta-analysis including data from four large phase III studies (CLASSIC, ARTIST, MAGIC, and ITACA-S trial) explored the role of adjuvant chemotherapy in the MSI-H subtype [29]. It showed that for resectable MSI-H/dMMR GC patients, the prognosis of patients who received surgery alone was better than those who underwent surgery followed by adjuvant chemotherapy, even though the sample size of MSI-H/dMMR in this meta-analysis was very modest (N = 121). Based on this result, adjuvant chemotherapy is not recommended for resectable MSI-H/dMMR GC patients in the latest ESMO guideline [5]. Additionally, the updated CSCO guidelines suggest that either observation or adjuvant chemotherapy could be considered after a thorough discussion with the patients regarding the possible risks and benefits [17].

### Adjuvant chemoradiotherapy

Unlike chemotherapy, the role of radiotherapy for resectable GC in the adjuvant setting is controversial. Adjuvant chemoradiotherapy (CRT) was once adopted in North America, according to the results of the phase III INT-0116 trial [30]. In this study, 556 patients with resectable GC or GEJ adenocarcinoma were randomly assigned to the upfront surgery plus adjuvant CRT group or the surgery group. Patients in the experimental arm received adjuvant fluorouracil chemotherapy plus 4500 cGy of radiation (5 × 5). Overall, CRT did prolong the OS compared to surgery alone (36 vs. 27 months, respectively; *P* = 0.005). However, most patients in this study received D0 or D1 lymphadenectomy and only 10% had D2 lymphadenectomy. The extent of dissection might affect the outcome of the surgery-only group. The phase III ARTIST trial from Korea evaluated the role of postoperative CRT based on the D2 dissection backbone [31]. Four hundred fifty-eight patients who received D2 lymphadenectomy and R0 resection were enrolled and randomly assigned to the adjuvant chemotherapy arm (capecitabine plus cisplatin, XP) or the adjuvant CRT arm (XP-XRT-XP). Unfortunately, the addition of radiotherapy postoperatively did not improve their DFS (*P* = 0.0862). However, in the subgroup analysis, DFS was significantly prolonged in the CRT arm in the patients with lymph node-positive (N +) disease (3-year DFS rate: 77.5% vs.72.3%, HR 0.69, 95% CI 0.474–0.995, *P* = 0.0365). Based on these findings, the subsequent ARTIST II trial further explored the role of adjuvant CRT in patients with lymph node-positive GC [32]. Five hundred forty-six patients after D2 dissection were randomly assigned to adjuvant S-1, adjuvant SOX, and adjuvant SOX plus radiotherapy (SOXRT) in a 1:1:1 ratio. However, there was no significant difference in DFS between the adjuvant SOX and SOXRT treatments (3-year DFS rate: 72.8% vs.74.3%; HR 0.97, 95% CI 0.66–1.42, *P* = 0.879). Therefore, according to current results from these clinical trials, adjuvant CRT is not recommended in patients who received D2 lymphadenectomy and R0 resection.

### Novel perioperative therapies

#### Perioperative targeted therapy

Anti-HER2 and anti-vascular endothelial growth factor (VEGF) therapies have been recommended as the standard treatments for advanced GC in the first- and second-line setting, respectively. However, the role of targeted therapy in the perioperative or adjuvant setting is still unclear and is currently under investigation.

##### Anti-HER2 therapy

According to the ToGA study, adding trastuzumab to chemotherapy improved the OS in patients with metastatic HER2-positive GC [14]. However, the role of anti-HER2 therapy in resectable GC was unclear. In the multicenter phase II HER-FLOT study, patients with HER2-positive esophagogastric adenocarcinoma received perioperative FLOT chemotherapy for four cycles preoperatively and four cycles postoperatively, followed by 9 cycles of trastuzumab monotherapy [33]. The pCR rate was 21.4%, and the median DFS was 42.5 months. The phase II randomized PETRARCA study investigated the efficacy of adding trastuzumab and pertuzumab to perioperative FLOT chemotherapy in patients with ≥ cT2 or cN + resectable GC [34]. The pCR rate was significantly improved with trastuzumab and pertuzumab (35% vs. 12%, *P* = 0.02), and the R0 resection rate and surgical morbidity were comparable between both groups. However, adding targeted therapy to perioperative chemotherapy did not improve DFS or OS and caused more severe adverse events (≥ grade 3), especially diarrhea (41% vs. 5%) and leukopenia (23% vs. 13%). Based on these results, the trial did not proceed to phase III. Another phase II NEOHX study recruited 36 HER2-positive GC patients who received perioperative CAPOX plus trastuzumab treatment, followed by 12 cycles of trastuzumab maintenance therapy [35]. The pCR rate, 18-month DFS rate, and 5-year OS rate were 9.6%, 71%, and 58%, respectively. The randomized phase II INNOVATION trial assigned patients to 3 groups: perioperative chemotherapy, chemotherapy plus trastuzumab, and chemotherapy plus trastuzumab and pertuzumab [36]. According to the investigators' choice, the chemotherapy could be FLOT, CAPOX, FOLFOX, or XP. The primary endpoint was major pathological response (MPR) rate, and the result is pending. In general, adding anti-HER2 therapy to chemotherapy showed certain efficacy in the perioperative setting, but the associated survival benefit should be further investigated in a larger randomized trial.

##### Anti-VEGF therapy

As for anti-VEGF therapy, the randomized, open-label, phase II/III ST03 trial recruited 1,063 resectable esophagogastric adenocarcinoma patients and randomly assigned them to perioperative chemotherapy (ECX) group or perioperative chemotherapy plus bevacizumab group [37]. The result showed that adding bevacizumab did not improve the 3-year OS (48.1% vs. 50.3% for chemotherapy alone; HR 1.08; 95% CI, 0.91 to 1.29; *P* = 0.36). Besides, adding bevacizumab was associated with higher rates of postoperative anastomotic leak (24% vs. 10%). Ramucirumab, a VEGF receptor 2 inhibitor, has become one of the standard choices in the second-line treatment of GC [5, 17, 18]. The RAMSES/FLOT7 evaluated the efficacy of adding ramucirumab to perioperative FLOT for resectable GC [38]. The R0 resection rate in the intervention group was improved compared to the chemotherapy group (96% vs. 82%, *P* = 0.0093). The median DFS was prolonged in the FLOT plus ramucirumab group (32 months vs. 21 months), while the OS was similar in both groups (46 months vs. 45 months).

#### Perioperative immunotherapy

Based on several phase III clinical trials, programmed death 1 (PD-1) inhibitors were approved for first- and third-line treatment of unresectable/metastatic GC in different countries [5, 17, 18]. However, the role of ICI in resectable GC remains unclear and is being investigated in various clinical trials. In the randomized phase II DANTE trial, patients with resectable GC were assigned to the experimental arm with the PD-L1 inhibitor atezolizumab plus FLOT chemotherapy and the control arm with standard FLOT chemotherapy [39]. The R0 resection rate, surgical morbidity and mortality were comparable in both groups. Atezolizumab combined with chemotherapy was associated with tumor downstage and pathological regression, which were more pronounced in patients with a higher PD-L1 combined positive score (CPS).

Several single-arm phase II clinical trials explored the efficacy of perioperative ICIs combined with different treatments (chemotherapy, targeted therapy, or radiotherapy) in resectable GC [40–44]. The pCR rates ranged from 10 to 41%. In the phase III ATTRACTION-5 trial (NCT03006705), the use of nivolumab in the adjuvant setting was investigated. Patients who have undergone D2 surgery will receive either S-1 for one year or CAPOX for six months, with nivolumab added to the adjuvant therapy in the intervention arm. The primary endpoint of the study is relapse-free survival (RFS). The result was announced recently. Unfortunately, the addition of nivolumab did not extend the RFS compared with adjuvant chemotherapy alone. Additionally, the role of pembrolizumab in combination with perioperative chemotherapy for resectable GC is being examined in the phase III clinical trial, KEYNOTE-585 [45]. The chemotherapy regimens under investigation are XP, FP, or FLOT, and the primary endpoints of the study are OS, event-free survival (EFS), and pCR rate. The potential survival benefits and efficacy of ICI are also being evaluated in the double-blind, randomized phase III MATTERHORN study, which is based on the FLOT backbone [46]. Patients with resectable GC will receive either perioperative FLOT or FLOT plus durvalumab (a PD-L1 antibody). The primary endpoint of the study is EFS, with secondary endpoints including OS and pCR rate.

For the dMMR/MSI-H subgroup, as discussed above, the value of chemotherapy was controversial. Considering dMMR/MSI-H is a predictive biomarker for immunotherapy in advanced GC, treatment with immune checkpoint inhibitors in the perioperative setting has the potential to improve the response rate and survival. The phase II GERCOR NEONIPIGA study evaluated the response rate and safety of the combination of neoadjuvant nivolumab and low-dose ipilimumab followed by adjuvant nivolumab in patients with dMMR/MSI-H locally advanced G/GEJ adenocarcinoma. Among 29 patients who underwent surgery, 17 (58.6%; 90% CI, 41.8–74.1) achieved pCR[47]. Similarly, the pCR rate of tremelimumab plus durvalumab was 60% in the neoadjuvant setting (cohort 1) in the phase II INFINITY study[48]. Based on these encouraging results, it is possible for patients who achieved pCR after neoadjuvant immunotherapy to avoid surgery. Cohort 2 of the INFINITY study has started enrollment to investigate the activity of tremelimumab plus durvalumab as the definitive treatment for dMMR/MSI-H locally advanced GC.

## Management for unresectable/metastatic GC

### Chemotherapy

Cytotoxic agents, including fluoropyrimidine, platinum, taxanes and irinotecan, are the main treatment in advanced gastric cancer. Generally, fluoropyrimidine (fluorouracil, capecitabine, and S-1) combined with platinum is used as the backbone therapy in the first line. Oxaliplatin is considered to be as effective as cisplatin. In the phase III SOX-GC trial, the SOX regimen showed improved survival compared to the SP regimen in diffuse or mixed-type GC[49]. For patients who are not fit for intensive chemotherapy (older age or poor performance status), the phase III GO2 trial showed that a modified dose of two-drug chemotherapy (60% of the full dose) provided a better tolerance but did not compromise the clinical outcome[50]. Paclitaxel, docetaxel, and irinotecan are commonly used in the second line of chemotherapy. In the ABSOLUTE phase III clinical trial conducted in Japan, weekly use of albumin-bound paclitaxel (nab-paclitaxel) was not inferior to weekly solvent-based paclitaxel in terms of overall survival[51]. In third-line treatment, trifluridine-tipiracil (TAS-102), an oral cytotoxic agent, has been proven in the phase III TAGS trial to improve overall survival compared with placebo (5.7 vs.3.6 months, HR 0.69, 95% CI 0.56–0.85)[52].

### Immune Checkpoint Inhibitors (ICIs) in unresectable/metastatic GC

Immune checkpoint inhibitors (ICIs) (monotherapy or combined with other treatments) have shown anti-tumor effects across a spectrum of solid tumors, including gastrointestinal tumors. Here, we present an overview of current evidence of ICI treatment in GC (Table 2) and discuss different predictive biomarkers for ICIs.

**Table 2:** Summary of the key phase III clinical trials of ICIs in metastatic GC

Therapy line	Trial	Agent	Experimental arm	Control arm	Primary endpoints	Result (Experimental vs. Control)	References
First line (HER2-negative)	KEYNOTE-062	Pembrolizumab	Pembrolizumab monotherapy; Pembrolizumab plus chemotherapy (PF or XP)	Chemotherapy (PF or XP)	OS and PFS in pts with CPS ≥ 1	Pembro vs. Chemo: OS: 10.6 vs. 11.1 months (HR, 0.91; 99.2% CI, 0.69–1.18); PFS: 2.0 vs. 6.4 months (HR, 1.66; 95% CI, 1.37–2.01); Pembro + chemo vs. Chemo: OS: 12.5 vs. 11.1 months (HR, 0.85; 95% CI, 0.70–1.03); PFS: 6.9 vs. 6.4 months (HR, 0.84; 95% CI, 0.70–1.02)	[53, 54]
	CheckMate-649	Nivolumab	Nivolumab plus chemotherapy (XELOX or FOLFOX)	Chemotherapy (XELOX or FOLFOX)	OS and PFS in pts with CPS ≥ 5	Nivo + chemo vs. Chemo: OS: 14.4 vs. 11.1 months (HR 0.71; 98.4% CI, 0.59–0.86); PFS: 7.7 vs. 6.05 months (HR 0.68; 98% CI, 0.56–0.81); Nivo + ipi vs. Chemo: OS: 11.2 vs. 11.6 months (HR, 0.89; 95% CI, 0.71–1.10); PFS: 2.8 vs. 6.3 months (HR, 1.42; 95% CI, 1.42–1.76)	[56, 86]
	ATTRACTION-4	Nivolumab	Nivolumab plus chemotherapy (SOX or CAPOX)	Chemotherapy (SOX or CAPOX)	OS and PFS	OS: 17.45 vs. 17.15 months (HR 0·90; 95% CI 0·75–1·08); PFS: 10.45 vs. 8.34 months (HR 0·68; 98·51% CI 0·51–0·90)	[58]
	ORIENT-16	Sintilimab	Sintilimab plus chemotherapy (CapeOX)	Chemotherapy (CapeOX)	OS in pts with CPS ≥ 5	OS: 18.4 vs. 12.9 months (HR 0.660; 95% CI 0.505–0.864);	[59]
	RATIONALE-305	Tislelizumab	Tislelizumab plus chemotherapy (XELOX or PF)	Chemotherapy (XELOX or PF)	OS and PFS	In the PD-L1 + (i.e., PD-L1 TAP score ≥ 5%) population: OS: 17.2 vs. 12.6 months (HR 0·74; 95% CI 0·59–0·94); PFS: 7.2 vs. 5.9 months (HR 0·67; 95% CI 0·55–0·83)	[60, 61]
	KEYNOTE-859	Pembrolizumab	Pembrolizumab plus chemotherapy (CAPOX or PF)	Chemotherapy (CAPOX or PF)	OS	OS: 12.9 vs. 11.5 months (HR 0·78; 95% CI 0·70–0·87); PFS: 6.9 vs. 5.6 months (HR 0·76; 95% CI 0·67–0·85)	[55]
	JAVELIN Gastric 100	Avelumab	Avelumab maintenance after 12 weeks of 1st-line chemotherapy (FOLFOX)	Continued chemotherapy (FOLFOX)	OS	OS: 10.4 vs. 10.9 months (HR 0.91; 95% CI, 0.74–1.11)	[60, 62]
First line (HER2-positive)	KEYNOTE-811	Pembrolizumab	Pembrolizumab plus trastuzumab and chemotherapy (XELOX or PF)	Trastuzumab plus chemotherapy (XELOX or PF)	OS and PFS	ORR (74.4% vs. 51.9%); results of PFS and OS are immature	[73]
Sencond line	KEYNOTE-061	Pembrolizumab	Pembrolizumab	Chemotherapy (paclitaxel)	OS and PFS in pts with CPS ≥ 1	OS: 9.1 vs. 8.3 months (HR 0·82; 95% CI 0·66–1·03); PFS: 1.5 vs. 4.1 months ((HR 1·27, 95% CI 1·03–1·57)	[84]
Third line	ATTRACTION-2	Nivolumab	Nivolumab	Placebo	OS	OS: 5.3 vs. 4.1 months (HR 0·63, 95% CI 0·51–0·78)	[64]
	JAVELIN Gastric 300	Avelumab	Avelumab	Chemotherapy (paclitaxel or irinotecan)	OS	OS: 4.6 vs. 5.0 months (HR 1.1, 95% CI 0·9–1.4)	[65]

#### First line

KEYNOTE-062 was the first global, randomized phase III trial to compare the efficacy and safety of immuno-monotherapy (pembrolizumab) or immunotherapy plus chemotherapy versus standard chemotherapy in HER2-negative advanced GC in the first-line setting [53]. According to the last update in ASCO 2022, it was suggested that pembrolizumab monotherapy was non-inferior to chemotherapy alone (cisplatin and fluorouracil/capecitabine) in patients with PD-L1 CPS ≥ 1 (median OS 10.6 vs. 11.1 months, HR 0.90, 95% CI 0.75–1.08) but was superior in the CPS ≥ 10 population (median OS 17.4 vs. 10.8 months; HR, 0.62; 95% CI, 0.45–0.86) [54]. However, the combination of pembrolizumab and chemotherapy did not bring OS benefit compared to chemotherapy alone in either CPS ≥ 1 (12.5 vs. 11.1 months; HR, 0.85; 95% CI, 0.71–1.02) or CPS ≥ 10 (12.3 vs. 10.8 months; HR, 0.76; 95% CI, 0.56–1.03) subgroup [54]. In another double-blind, placebo-controlled phase III KEYNOTE-859 study, the addition of pembrolizumab to chemotherapy (FP or CAPOX) demonstrated slight survival benefit compared with chemotherapy alone (OS 12.9 vs. 11.5 months, HR, 0.78; 95% CI, 0.70–0.87. PFS 6.9 vs. 5.6 months, HR, 0.76; 95% CI, 0.67–0.85). The results were generally consistent in different PD-L1 CPS subgroups[55].

CheckMate-649 is another global, randomized, phase III trial investigating the effects of ICIs (nivolumab plus ipilimumab, a CTLA-4 inhibitor) or ICI (nivolumab) plus chemotherapy versus chemotherapy (CAPOX or FOLFOX) alone in metastatic HER2-negative GC patients [56]. One thousand five hundred eighty-one patients were assigned to nivolumab plus chemotherapy arm or chemotherapy arm. The addition of nivolumab to chemotherapy improved the OS (14.4 vs. 11.1 months; HR 0.71; 98.4% CI, 0.59 to 0.86; P < 0.0001) and PFS (7.7 vs. 6.05 months; HR 0.68; 98% CI, 0.56 to 0.81; *P* < 0.0001) for the patients with PD-L1 CPS ≥ 5; therefore both primary endpoints were met. For all-randomized patients, nivolumab combined with chemotherapy also improved OS (13.8 vs. 11.6 months; HR 0.80; 99.3% CI, 0.68 to 0.94; *P* = 0.0002). Moreover, all CPS subgroups exhibited an increased objective response rate in the nivo-chemotherapy arm. However, the chemo-free treatment with nivolumab and ipilimumab did not show OS improvement compared to chemotherapy alone [57]. Based on these findings, nivolumab combined with chemotherapy was listed as one of the recommended first-line treatments in the NCCN, ESMO, and CSCO guidelines [5, 17, 18].

ATTRACTION-04 was a randomized, double-blind, placebo-controlled, multicenter phase II/III trial that evaluated the effects of nivolumab plus chemotherapy (SOX or CAPOX) compared with chemotherapy alone in the first-line treatment for HER2-negative advanced GC in the Asian population, regardless of PD-L1 expression [58]. The combination therapy significantly improved the PFS (HR 0·68; 98·51% CI 0·51–0·90; *P* = 0·0007) but not the OS (both groups achieved > 17 months of median OS). One of the possible reasons for the different results of OS between ATTRACTION-04 and CheckMate-649 could be the subsequent anticancer therapies, whereby the proportion of patients who received subsequent anticancer treatments or ICIs therapy was much higher in ATTRACTION-04 (66% vs. 39% in CheckMate-649).

The efficacy of immunotherapy plus chemotherapy was further confirmed in the Asian phase III ORIENT-16 trial, which compared sintilimab plus chemotherapy (CAPOX) to chemotherapy alone as the first-line treatment [59]. The pre-specified interim result was reported at ESMO 2021. Sintilimab plus chemotherapy showed a survival benefit versus chemotherapy alone in patients with CPS ≥ 5 (18.4 vs. 12.9 months; HR 0.660; 95% CI 0.505–0.864) and all randomized patients (15.2 vs. 12.3 months; HR 0.766; 95% CI 0.626–0.936). Another PD-1 antibody, tislelizumab, is currently being investigated in the phase III RATIONALE-305 trial [60]. Advanced GC patients are randomized to receive tislelizumab plus chemotherapy (CAPOX/FP regimen) or chemotherapy alone. The primary endpoints are PFS and OS. Results from the interim analysis of the PD-L1 + (i.e., PD-L1 TAP score ≥ 5%) population were represented at 2023 ASCO-GI, showing that tislelizumab plus chemotherapy led to significant OS (17.2 vs. 12.6 months; HR 0·74; 95% CI 0·59–0·94) and PFS (7.2 vs. 5.9 months; HR 0·67; 95% CI 0·55–0·83) improvement compared to chemotherapy alone[61]. The ITT population outcomes will be reported after the final analysis.

In summary, in first-line treatment for HER2-negative advanced GC, the addition of anti-PD-1 therapy could improve clinical outcomes in patients with high PD-L1 expression, according to the results from CheckMate-649, ORIENT-16, and RATIONALE-305. For patients with low PD-L1 expression or unknown PD-L1 status, the survival benefit of adding PD-1 antibodies is still controversial (discussed below), and the risk–benefit balance of ICIs treatment should be considered, and decisions should be discussed case by case.

The role of maintenance therapy with ICIs after first-line chemotherapy was evaluated in the phase III JAVELIN Gastric 100 trial [62]. Patients with HER2-negative advanced GC without progression after at least 12 weeks of first-line chemotherapy (oxaliplatin plus fluoropyrimidine) were randomly assigned to avelumab (a PD-L1 inhibitor) maintenance or continued chemotherapy. Avelumab maintenance did not show OS benefit compared to chemotherapy (24-month OS rate: 22.1% versus 15.5%; HR 0.91; 95% CI, 0.74–1.11; *P* = 0.1779).

#### Second line and beyond

The randomized, open-label, phase III KEYNOTE-061 trial compared pembrolizumab monotherapy with paclitaxel in patients with advanced GC or GEJ cancer in the second-line setting [53]. Though the primary endpoints (the OS and PFS in patients with PD-L1 CPS ≥ 1) were not met, it was suggested that the efficacy of pembrolizumab monotherapy was associated with the PD-L1 CPS level. Patients with CPS ≥ 10 had a better outcome in the pembrolizumab group than in the chemotherapy group.

KEYNOTE-059 was a phase II study that explored the effect of pembrolizumab in patients with advanced GC after progression from ≥ 2 lines of treatment [63]. Among the 259 patients enrolled, the ORR and median duration of response (DoR) was 11.6% and 8.4 months, respectively. Moreover, pembrolizumab showed higher efficacy in the subgroup with PD-L1-positive cancer (CPS ≥ 1) compared to PD-L1-negative cancers (ORR 15.5% vs. 6.4%; DoR 16.3 vs. 6.9 months, respectively). The phase III ATTRACTION-2 study compared nivolumab monotherapy versus placebo in advanced GC patients after two lines of therapy, regardless of the PD-L1 expression [64], and survival benefit was observed in the nivolumab group (OS 5.3 vs. 4.1 months; HR 0·63, 95% CI 0·51–0·78; *P* < 0·0001). Based on the results of this study, nivolumab is recommended as monotherapy in third-line treatment for GC in the CSCO guideline but not in the ESMO or NCCN guidelines due to the patients enrolled being exclusively Asian. The role of avelumab in the third-line treatment for advanced GC was investigated in the phase III JAVELIN Gastric 300 trial [65]. Though avelumab showed a more manageable safety than the physician's choice of chemotherapy, it did not improve OS (primary endpoint, 4.6 vs. 5.0 months; HR 1.1, 95% CI 0·9–1.4; *P* = 0.81), PFS, or ORR.

#### Molecular Biomarkers of Immunotherapy in GC

##### HER2

HER2-positive GC, defined as immunohistochemical (IHC) expression 3+ or 2 + combined with positive fluorescent in situ hybridization (FISH) verification, accounts for approximately 15–20% of gastric or gastroesophageal cancer. The phase III ToGA study has established trastuzumab combined with chemotherapy as the standard first-line treatment for HER2-positive advanced GC [14]. In preclinical models, HER2 signaling could regulate the recruitment and activation of tumor-infiltrating immune cells [66]. Besides, trastuzumab has been shown to upregulate the expression of PD-1 and PD-L1 [67, 68], and anti-PD-1 antibodies could significantly increase the therapeutic activity of HER2 inhibitors [69]. Several phase I/II studies demonstrated the promising efficacy of the addition of ICIs to trastuzumab and chemotherapy in HER2-positive GC. In the phase Ib Ni-HIGH study conducted in Japan, patients with HER2-positive advanced GC received nivolumab, trastuzumab, and chemotherapy (CAPOX or SOX regimen) in the first-line setting, and the ORR was 75%, as reported at ASCO 2020 [70]. The multi-institutional phase Ib/II PANTHERA trial explored the efficacy and safety of the combination of pembrolizumab, trastuzumab and chemotherapy as first-line therapy for HER2-positive advanced GC [71]. The updated data at ASCO-GI 2021 showed that the ORR was 76.7% (CR 16.3%, PR 60.5%), the PFS was 8.6 months (95% CI 7.2–16.5 months), and the OS was 19.3 months (95% CI 16.5-NR). The striking efficacy was also reported in another phase II study, in which patients with HER2-positive GC received pembrolizumab, trastuzumab and chemotherapy (oxaliplatin/cisplatin + capecitabine/5-FU) [72]. Overall, the ORR was 91% and DCR was 100%. The median PFS and OS was 13·0 months and 27·3 months, respectively, which was much better than the OS reported in the ToGA study. Recently, the randomized, double-blind, placebo-controlled phase III KEYNOTE-811 trial reported the results of its first interim analysis [73], in which patients with metastatic HER2-positive GC or GEJ cancer received pembrolizumab or placebo plus trastuzumab and chemotherapy. The results showed that adding pembrolizumab to trastuzumab and chemotherapy could markedly increase the ORR (74.4% vs. 51.9%; the estimated difference between the two groups was 22.7%; 95% CI, 11.2–33.7%; *P* = 0.00006). Based on this result, the FDA approved pembrolizumab combined with trastuzumab and chemotherapy as the first-line treatment for advanced HER2-positive gastric or GEJ adenocarcinoma. The results of the primary endpoints (PFS and OS) are still immature.

##### MSI

MSI-H tumor is one of the four subtypes of GC according to The Cancer Genome Atlas (TCGA) Research Network [11]. The incidence of MSI-H status in GC was reported to range from 8 to 25%, which was much lower in metastatic disease [74]. Mismatch repair (MMR) proteins are supposed to fix the errors that occur during DNA replication. When MMR proteins are deficient, the defects of DNA replication will lead to the accumulation of mutations and the expression of neoantigens, which may act as potential targets of immune cells [75]. Hence, it is reasonable that tumors with MSI-H/dMMR status may attract more immune cell infiltration and enhance the effect of immune checkpoint inhibitors. A post hoc analysis of KEYNOTE-059 (third-line treatment), KEYNOTE-061 (second-line treatment), and KEYNOTE-062 (first-line treatment) was conducted to evaluate the efficacy of pembrolizumab versus chemotherapy in the patients with MSI-H advanced G/GEJ adenocarcinoma [15]. Overall, 7 of 174 patients (4.0%) in KEYNOTE-059, 27 of 514 (5.3%) in KEYNOTE-061, and 50 of 682 (7.3%) in KEYNOTE-062 with MSI-H status were enrolled. By the time of analysis, the OS of the patients with MSI-H was not reached for pembrolizumab monotherapy in KEYNOTE-059, 061 and 062, or for pembrolizumab combined with chemotherapy in KEYNOTE-062, compared with an OS of around 8 months for chemotherapy alone. Besides, the ORR was much higher in the immunotherapy groups. In another meta-analysis including four phase III trials (KEYNOTE-062, CheckMate-649, JAVELIN Gastric 100, and KEYNOTE-061), 2545 patients with known MSI status were enrolled, and the proportion of MSI-H was 4.8% [76]. In the MSI-H group, the HR for OS benefit with immunotherapy was 0.34 (95% CI 0.21–0.54), compared to 0.85 (95% CI 0.71–1.00) for the MSS group. Among the patients with MSI-H status, the HR for PFS was 0.57 (95% CI 0.33–0.97; *P* = 0.04), and the odds ratio (OR) for ORR was 1.76 (95% CI 1.10–2.83; *P* = 0.02). Altogether, these findings suggested that MSI-H status was a predictive biomarker for immune checkpoint inhibitor treatments, regardless of the line of therapy.

##### EBV

Epstein-Barr virus-associated GC (EBVaGC) is another distinct molecular subtype of the TCGA classification [11], accounting for about 9% of GC in the TCGA cohort and approximately 5% in China [77, 78]. EBV has been linked to CD8^+^ T cell infiltration and increased expression of PD-L1 and PD-L2 [11, 79], making it a potential biomarker for ICI treatment. While a Korean study with a small sample size (n = 6) once reported a 100% response rate in EBV-positive advanced GC [80], several other studies did not demonstrate a high response rate [81–83]. Differences in response rates across studies may be attributed to confounding factors such as tumor mutational burden (TMB) and PD-L1 expression. Therefore, the role of EBV positivity in immunotherapy for GC remains unclear and requires further investigation.

##### PD-L1

As discussed earlier, the level of PL-L1 expression, especially the CPS score, has been considered a predictive biomarker for response to ICIs. However, the reliable cut-off value to predict the benefit of immunotherapy is needed to be determined. The cut-off points often used in clinical trials are 1, 5 and 10. In the KEYNOTE-059 trial, CPS ≥ 1 was used to separate the patients that could benefit from third-line pembrolizumab treatment [63]. However, this benefit was not seen compared to chemotherapy in the KEYNOTE-061/062 trials [53, 84]. In KEYNOTE-061/062, CPS ≥ 10 effectively differentiated the response to pembrolizumab. Patients with CPS ≥ 10 had better OS benefits than those with CPS ≥ 1. A comprehensive analysis of patients with CPS ≥ 10 in KEYNOTE-059, 061 and 062 also showed consistent improvement toward better outcomes with pembrolizumab in different lines of treatment in this subgroup [85]. In the CheckMate-649 and ORIENT-16 studies, CPS ≥ 5 was used as the cut-off value for the primary endpoint OS. Though the OS benefit of nivolumab plus chemotherapy was also observed in all randomized patients in CheckMate-649, the subgroup analysis suggested that the benefit was insignificant in the CPS < 5 or < 1 group [86]. A recent study reconstructed unreported Kaplan–Meier plots of PD-L1 CPS subgroups of three phase III trials (CheckMate-649, KEYNOTE-062, and KEYNOTE-590) and investigated the outcome of low CPS subgroup [87]. The result suggested that patients with low PD-L1 expression (CPS 1–4 and CPS 1–9) did not benefit from adding ICIs to chemotherapy. In summary, although the predictive role of PD-L1 CPS for immunotherapy efficacy has been demonstrated in multiple clinical trials, there is still a need to determine the optimal cut-off value for CPS and to develop further classifications for patients with low CPS scores. Recently, the result of the phase III RATIONALE-305 trial suggested that the TAP score > 5% also had predictive value for ICI treatment in gastric cancer[61], and further exploration is needed.

##### Tumor mutation burden (TMB)

It is hypothesized that a high TMB status results in the high expression of neoantigens, which are immunogenic and can induce the response of the immune system and potentially increase the efficacy of ICI treatment. In a phase Ib/II study that explored the efficacy of the PD-1 antibody toripalimab in patients with advanced GC, patients with TMB-high (TMB-H, TMB ≥ 12 mut/Mb) showed a higher ORR and better OS compared with patients with TMB-L status (ORR 33.3% vs. 7.1%, *P* = 0.017; OS 14.6 vs. 4.0 months, *P* = 0.038)[88]. In the subgroup analysis of the KEYNOTE-061 study, the TMB status (≥ 10 or < 10 mut/Mb) was associated with response rate, PFS, and OS in patients treated with pembrolizumab. In the TMB-H subgroup, pembrolizumab demonstrated a better OS compared with paclitaxel, and this benefit remained even when MSI-H patients were excluded[89]. Though FDA granted approval for the use of pembrolizumab in patients with TMB-H (i.e., TMB ≥ 10 mutations/Mb) advanced solid tumors that progressed after standard treatments, according to the subgroup analysis of KEYNOTE-158 study[90], the evidence is still not enough for the use of ICIs in TMB-H gastric cancer, and phase III studies to illustrate the predictive value of TMB are needed.

### Molecular targeted therapy in unresectable/metastatic GC

Molecular targeted therapy remains an essential treatment option for patients with advanced GC, aimed to inhibit tumor proliferation and increase survival rates. Targeted therapies, including anti-HER2, anti-angiogenesis, and other biomarker-directed therapies, have demonstrated promising efficacy in treating GC, with significant benefits observed in biomarker-enriched patients (Table 3). Therefore, next-generation sequencing or ctDNA detection is crucial for mGC patients to establish a comprehensive molecular profile, including the status of HER2, fibroblast growth factor receptor (FGFR), Claudin18.2 (CLDN18.2), PD-L1 and EGFR.

**Table 3:** Selected key clinical trials of targeted therapies for mGC patients

Trial	Phase	Treatment	Outcomes	References
**HER2**				
ToGA	III	Capecitabine or 5- FU plus cisplatin with vs without trastuzumab as first-line therapy	ORR 47% vs. 35%, * P* = 0.0017; mOS 13.8 vs. 11.1 months (HR 0.74, 95% CI 0.60–0.91; * P* = 0.0046); mPFS 6.7 vs 5.5 months (HR 0.71, 95% CI 0.59–0.85; * P* = 0.0002)	[14]
DESTINY-Gastric01	II	Trastuzumab deruxtecan vs chemotherapy (Irinotecan or Paclitaxel) as third or later-line therapy	ORR 51% vs. 14%, *P* < 0.001; mOS 12.5 vs. 8.4 months (HR 0.59; 95% CI, 0.39 to 0.88; * P* = 0.01); mPFS 5.6 vs. 3.5 months (HR 0.47; 95% CI, 0.31 to 0.71)	[132]
DESTINY-Gastric02	II	Trastuzumab deruxtecan as second-line therapy	ORR 41.8%, mPFS 5.6 months (95% CI 4.2–8.3 months); mOS 12.1 months (95% CI 9.4–15.4 months)	[134]
RC48-C008	II	RC48 as third-line therapy and beyond	ORR 24.8% (95% CI 17.5%-33.3%); mPFS 4.1 months (95% CI 3.7–4.9 months); mOS 7.9 months (95% CI 6.7–9.9 months)	[137]
**VEGF/ VEGFR**				
RAINBOW	III	Paclitaxel with vs without ramucirumab as second-line therapy	ORR 28% vs. 16%, *P* = 0.0001; mOS 9.6 vs 7.4 months (HR 0.81, 95% CI 0.68–0.96; *P* = 0.017); mPFS 4.4 vs. 2.9 months (HR 0.64, 95% CI 0.54–0.75; *P* = 0.0001)	[145]
REGARD	III	Ramucirumab vs placebo as second-line therapy	ORR 3% vs. 3%, *P* = 0.76; mOS 5.2 vs. 3.8 months (HR 0.78, 95% CI 0.61–0.1; *P* = 0.047); mPFS 2.1 vs. 1.3 months (HR 0.48, 95% CI 0.38–0.62; *P* < 0.0001)	[144]
RAINBOW-Asia	III	Ramucirumab plus paclitaxel vs placebo plus paclitaxel as second-line therapy	mPFS 4.14 vs 3.15 months (HR 0.765, 95% CI 0.613–0.955, *P* = 0.0184); mOS 8.71 vs 7.92 months (HR 0.963, 95% CI 0.771–1.203, *P* = 0.7426)	[146]
Li et al	III	Apatinib vs placebo as third or later-line therapy	ORR 2.84% vs. 0.00%, *P* = 0.17; mOS 6.5 vs. 4.7 months (HR 0.71, 95% CI 0.54–0.94; *P* = 0.015); mPFS 2.6 vs. 1.8 months (HR 0.44, 95% CI 0.33–0.6; *P* < 0.001)	[152]
ANGEL	III	Apatinib + BSC vs placebo + BSC as third or later-line therapy	In ≥ 3rd-line patients: mOS 5.78 vs. 5.13 months (HR = 0.93; 95% CI 0.74–1.15; *P* = 0.4850); mPFS 2.83 vs. 1.77 months (HR = 0.57; 95% CI 0.46–0.79; *P* < 0.0001); ORR 6.87% vs. 0%, *P* = 0.0020; DCR 42.37% vs. 13.08%, *P* < 0.0001 In ≥ 4th-line patients: mOS 6.43 vs. 4.73 months (HR = 0.65; 95% CI 0.46–0.92; *P* = 0.0195); mPFS 3.52 vs 1.71 moths (HR = 0.38; 95% CI 0.27–0.53; *P* < 0.0001)	[153]
**CLDN18.2**				
SPOTLIGHT	III	Zolbetuximab + mFOLFOX6 vs placebo + mFOLFOX6 as first-line therapy in patients with CLDN18.2-positive and HER-2-negative advanced gastric or GEJ cancer	mPFS 10.61 vs. 8.67 months (HR 0.751, *P* = 0.0066) mOS 18.23 vs. 15.54 months (HR 0.750, *P* = 0.0053)	[162]
GLOW	III	Zolbetuximab + CAPOX vs placebo + CAPOX as first-line therapy in patients with CLDN18.2-positive and HER-2-negative advanced gastric or GEJ cancer	mPFS (8.21 vs 6.80 months, HR 0.687, *P* = 0.0007) and mOS (14.39 vs. 12.16 months, HR 0.771, *P* = 0.0118)	[163]
**FGFR**				
FIGHT	II	Bemarituzumab + mFOLFOX6 vs placebo + mFOLFOX6 as first-line therapy	ORR 47% vs. 33%; mOS not reached vs. 12.9 months (HR 0.58, 95% CI 0.35–0.95; *P* = 0.027); mPFS 9.5 vs. 7.4 months (HR 0.68, 95% CI 0.44–1.04; *P* = 0.073)	[172]

5- FU, 5- fluorouracil; HR, hazard ratio; mOS, median overall survival; mPFS, median progression- free survival; ORR, objective response rate; DCR, disease control rate; mDoR, median duration of response; CI, confidence interval; NE, not evaluable; BSC, best supportive care

#### Anti-HER2 therapy

HER2, also known as ERBB2, is a member of the ERBB protein families that includes the epidermal growth factor receptor (EGFR or HER1), HER3, and HER4 [91]. HER2 overexpression or amplification has been found in a range of 7.3% to 20.2% in advanced gastric and gastroesophageal junction adenocarcinomas, with the overexpression rate varying globally [92]. In addition, intestinal-type gastric cancers and those arising from the proximal stomach or gastroesophageal junction are more likely to exhibit HER2 positivity. [11, 93].

Trastuzumab is a humanized monoclonal antibody that targets HER2 extracellular domain 4, then inhibits downstream signal activation and cancer cell proliferation. Trastuzumab plus chemotherapy has been established as the standard first-line treatment for HER2-positive advanced GC. The landmark ToGA trial revealed that trastuzumab plus chemotherapy significantly improved the overall survival of patients with advanced GC [14], especially for patients with HER2 positivity, who were identified as having HER2 immunohistochemistry (IHC) scores of 2 + and fluorescence in situ hybridization (FISH)-positive or HER2 IHC 3 + based on a post-hoc exploratory analysis [92]. The EVIDENCE trial has demonstrated that combining first-line trastuzumab with chemotherapy was associated with improved clinical outcomes in Chinese patients with HER2-positive metastatic GC, providing real-world evidence. [94].

However, subsequent attempts of HER2-targeted therapy in advanced GC were not as successful as expected. Even though pertuzumab [95, 96], trastuzumab emtansine (T-DM1) [97], and lapatinib [98, 99] were all investigated in several first-line and second-line trials, no survival improvement was observed in any of these trials. Additionally, trastuzumab beyond progression also failed to show a survival benefit in pre-treated HER2-positive GC patients in the T-ACT trial [100].

##### Potential resistance mechanisms of HER2-targeted therapy

Primary or acquired resistance is a major impediment to the management of mGC patients, while mechanisms underlying the poor efficacy of HER2-directed therapy in GC are not fully understood. Multiple potential resistance mechanisms have been researched, as listed below, and further studies are warranted to improve treatment resistance in GC patients treated with HER2-targeted therapy in clinical settings.

***HER2 heterogeneity***

Intratumoral HER2 heterogeneity is observed in 23% to 79% of GC patients and is associated with patients’ survival [101–103]. Specifically, Shusuke et al. reported prolonged survival in homo-HER2 positive GC patients, defined as all tumor cells overexpressing HER2 in biopsy specimens [101]. Tumor cells with HER2 overexpression or amplification are killed during HER2-targeted therapy, while residual drug-resistant colonies keep proliferating and eventually take control, leading to tumor recurrence. As a result, resistance to HER2-targeted therapy has been associated with the heterogeneity of HER2 expression [101, 104, 105]. Discordance between next-generation sequencing and FISH/IHC may also indicate intratumoral heterogeneity and result in an unfavorable treatment outcome. In addition, there still exist discrepancies in HER2 status between primary tumor and metastatic sites, which increases the risk of HER2-targeted therapy failure due to false-positive HER2 detection [106, 107].

***Loss of HER2 expression***

For mGC patients experiencing progression on trastuzumab, 29–69% of them may experience loss of HER2 expression, which is an important factor responsible for resistance [108–110]. Given the risk of HER2 expression loss during treatment, patients should re-evaluate HER2 status upon progression after anti-HER2 therapy to determine the most optimal treatment.

***Gene amplification***

Receptor tyrosine kinase (RTK) amplification was commonly detected in MET-amplified metastatic GC, with 40% to 50% of cases exhibiting co-amplification of either HER2 or EGFR. These patients did not usually respond to HER2-targeted therapy, but MET and HER2 combination inhibition could sometimes bring extra clinical benefit [111]. CCNE1, which encodes the cell cycle regulator cyclin E1, is another oncogene co-amplified with HER2 in metastatic GC. CCNE1 co-amplification has been found to be more strongly related to HER2-positive AGC than to HER2-positive breast cancer [112]. In a phase II study of lapatinib with capecitabine and oxaliplatin in HER2-positive AGC patients, CCNE1 amplification was demonstrated to play a role in resistance to HER2-targeted therapy [113]. A high level of copy number variation for CCNE1 has also been associated with worse survival in patients with HER2-positive metastatic GC treated with trastuzumab [114]. Other studies have also reported that deletion of ErbB2 16 exon and co-mutation and/or amplification of KRAS, HER3, EGFR, PI3K or PTEN could contribute to the resistance of anti-HER2 therapy [109, 113, 115, 116].

***Alterations in intracellular signaling***

HER2-targeted therapy suppresses downstream signaling pathways by blocking the binding of HER2 receptors and ligands, which inhibits the migration and proliferation of tumor cells and leads to apoptosis. RTK/RAS/PI3K signaling alterations have been shown to be involved in the development of resistance to trastuzumab. [109]. Furthermore, activation of the bypass pathway might also result in resistance. Sampera et al. discovered that SRC-mediated persistent activation of the MAPK-ERK and PI3K-mTOR pathways was connected to the treatment resistance in HER2-positive GC cell lines [117]. NRF2 has also been associated with HER2 resistance by activating the PI3K-mTOR signaling pathway [118].

##### Newer HER2-targeted agents

To overcome intrinsic and acquired resistance to trastuzumab, various clinical trials have explored newer agents and combinations. The following innovative HER2-targeted agents for advanced metastatic GC are currently under investigation (Table 4): monoclonal antibodies (mAbs) (e.g., margetuximab), bispecific antibodies (BsAbs) (e.g., ZW25, KN026), antibody–drug conjugates (ADCs) (e.g., T-DXd, Disitamab vedotin, ARX788), tyrosine kinase inhibitors (TKIs) (e.g., tucatinib), and other novel therapeutic approaches.

**Table 4:** Selected investigational HER2–targeted agents for mGC

Approach	Agents	Trial	Phase	Lines	Treatment	Outcomes	References
Monoclonal antibody	Margetuximab	MAHOGANY	II/III	1st line	Margetuximab ± PD-1 inhibitor ± chemotherapy ± dual checkpoint inhibitor	Cohort A (margetuximab plus retifanlimab): ORR 53% (95% CI 36.1–68.5); DCR 73% (95% CI 56.1–85.4); mPFS 6.4 months (95% CI 6.0-NE); mOS not reached	[123]
Bispecific antibodies	Zanidatamab (ZW25)	Ku, G., et al	II	1st line	ZW25 + chemotherapy (CAPOX or FP)	ORR 75%; mDOR 16.4 months; mPFS 12.0 months	[124]
		HERIZON-GEA-01	III	1st line	ZW25 + chemotherapy with or without tislelizumab vs. trastuzumab + chemotherapy	Ongoing	[125]
	KN026	Xu et al	II	2nd or later lines	KN026	Cohort 1(HER2 IHC3 + or IHC 2 + ISH +): ORR 56% (95% CI 35%-76%); mDoR 9.7 months (95% CI 4.2- NE); mPFS 8.3 months (95% CI 4.2–11.4); mOS 16.3 months (95% CI 11.0- NE) Cohort 2 (HER2 IHC 1 + /2 + ISH- or IHC 0/1 + ISH +): ORR 14% (95% CI 2%-43%); mDoR 6.2 months (95% CI 3.2-NE); mPFS 1.4 months (95% CI 1.4–4.1); mOS 9.6 months (95% CI 3.5–14.9)	[127]
	PRS-343	-	II	2nd or later lines	PRS-343 + ramucirumab + paclitaxel; PRS-343 + tucatinib	Ongoing	NCT05190445
ADC	Trastuzumab deruxtecan (T-Dxd)	DESTINY-Gastric01	II	3rd or later lines	T-Dxd vs. chemotherapy (Irinotecan or Paclitaxel)	ORR 51% vs. 14%, *P* < 0.001; mOS 12.5 vs. 8.4 months (HR 0.59; 95% CI, 0.39 to 0.88; *P* = 0.01); mPFS 5.6 vs. 3.5 months (HR 0.47; 95% CI, 0.31 to 0.71)	[132]
		DESTINY-Gastric02	II	2nd line	T-Dxd	ORR 41.8%, mPFS 5.6 months (95% CI 4.2–8.3 months); mOS 12.1 months (95% CI 9.4–15.4 months)	[134]
	RC48	RC48-C008	II	3rd or later lines	RC48	ORR 24.8% (95% CI 17.5%-33.3%); mPFS 4.1 months (95% CI 3.7–4.9 months); mOS 7.9 months (95% CI 6.7–9.9 months)	[137]
	ARX788	Zhang, Y., et al	I	2nd or later lines	ARX788	ORR 37.9% (95% CI 20.7%-57.7%); DCR 55.2% (95% CI 35.7%-73.6%); mPFS 4.1 months (95% CI 1.4-6.4 months); mOS 10.7 months (95% CI 4.8-not reached)	[138]
TKI	Tucatinib	MOUNTAINEER-02	II/III	2nd-line	Phase II: Tucatinib + trastuzumab + ramucirumab + paclitaxel Phase III: Arm A: Tucatinib + trastuzumab + ramucirumab + paclitaxel Arm B: Placebo + ramucirumab + paclitaxel Arm C: Tucatinib + ramucirumab + paclitaxel	Ongoing	NCT04499924

##### Monoclonal antibodies

**Margetuximab**

Margetuximab is an Fc-engineered anti-HER2 mAb that targets the same epitope as trastuzumab but with a higher affinity for single-nucleotide polymorphisms of the activating Fc receptor (CD16A) [119, 120]. Margetuximab can recruit CD16A-expressing natural killer cells, macrophages and monocytes and further promote antibody-dependent cell-mediated cytotoxicity (ADCC) [119]. The first phase I study of margetuximab in humans illustrated that margetuximab was well-tolerated with promising efficacy in relapsed HER2-overexpressing carcinoma [121]. Later in the phase Ib/II CP-MGAH22-05 study, patients with previously treated HER2-positive GC responded effectively to a chemotherapy-free treatment consisting of margetuximab plus pembrolizumab. Patients with HER2 IHC3 + and PD-L1 positive (CPS ≥ 1, by IHC) had an ORR of 44% and a DCR of 72% [122]. More recently, the phase II/III MAHOGANY trial has reported the efficacy of margetuximab plus anti-PD-1 antibody retifanlimab (Cohort A) for the first-line treatment of patients with G/GEJ adenocarcinoma, with an ORR and a DCR of 53% and 73% [123]. The ORR reported in this trial was superior to the ORR observed with other history chemotherapy-free treatments; nonetheless, given that chemotherapy-based regimens remain the predominant treatment for GC, the MAHOGANY trial has been halted for commercial reasons.

##### Bispecific antibodies (BsAbs)

**Zanidatamab (ZW25)**

Zanidatamab (ZW25) is a novel HER2-targeted bispecific antibody that binds to HER2 extracellular domain (ECD) II and IV. According to a phase I study, ZW25 was well tolerated with durable response in heavily pretreated GEA patients (including prior HER2-targeted therapy) [86]. Later in a phase II trial involving patients with advanced/metastatic HER2-positive GEA, zanidatamab plus chemotherapy (CAPOX or FP) showed a confirmed ORR of 75%, mDOR of 16.4 months and mPFS of 12.0 months in the first-line setting [124]. Based on these findings, a global phase III study (HERIZON-GEA-01) has been designed to assess the efficacy and safety profiles of zanidatamab plus chemotherapy with or without tislelizumab versus standard of care (trastuzumab plus chemotherapy) for patients with metastatic HER2-positive GEAs in first-line settings [125].

**KN026**

KN026 mimics the dual effects of trastuzumab and pertuzumab by simultaneously binding to HER2 ECD II and IV [126]. In a phase II clinical study, KN026 showed favorable results in patients with HER2-overexpressing G/GEJ adenocarcinoma (IHC3 + or IHC 2 + ISH +) with an ORR of 56% [127]. The ongoing phase II/III trial (KN026-001) is planned to evaluate the survival benefit of KN026 plus chemotherapy in patients with HER2-positive unresectable or advanced G/GEJ adenocarcinoma upon progression after trastuzumab-containing treatment (NCT05427383). Most recently, the preliminary data presented at ESMO 2022 illustrated that KN026 plus KN046, a recombinant humanized PD-L1/CTLA-4 bispecific antibody, had remarkable efficacy and tolerable safety in HER2-positive G/GEJ patients without prior systemic treatment [128]. In this phase II study, the ORR was 77.8%, and the DCR was 92.6%, indicating the need for a future randomized clinical trial to confirm the efficacy of KN026 plus KN046 treatment versus standard of care.

**Other BsAbs**

PRS-343 is a BsAb that targets HER2 and the costimulatory immunoreceptor 4-1BB on immune cells. In patients with advanced HER2-positive solid tumors, including GC, PRS-343 showed anticancer efficacy both alone and in combination with the anti-PD-L1 antibody atezolizumab in a phase I clinical study [129]. A phase II study (NCT05190445) is ongoing to investigate the efficacy of PRS-343 in combination with ramucirumab and paclitaxel in patients who have already received treatment for HER2-high (IHC 3+ or IHC 2+ with HER2/neu gene amplification) G/GEJ adenocarcinoma and in combination with tucatinib in HER2-low (IHC 1+ or IHC 2+ without HER2/neu gene amplification) G/GEJ adenocarcinoma.

##### Antibody–drug conjugates (ADCs)

**Trastuzumab deruxtecan (T-DXd)**

Trastuzumab deruxtecan (T-DXd) is an antibody–drug conjugate (ADC) composed of an anti-HER2 antibody connected to a cytotoxic topoisomerase I inhibitor via a cleavable tetrapeptide-based linker [130]. Different from T-DM1, T-DXd has a bystander effect on nearby cells, including those not expressing HER2, thus greatly enhancing the antitumor effect [131]. This action method is inspiring, particularly for advanced GC patients with diverse intratumoral HER2 expression. In the Asia DESTINY-Gastric01 trial, T-DXd significantly improved overall survival in patients with HER2 + advanced GC compared with chemotherapy in the later-line settings [132]. Interestingly, the efficacy and safety of T-DXd were also evaluated in exploratory cohorts of patients with HER2-low G/GEJ cancers in the DESTINY-Gastric01 trial (cohort 1, IHC 2 + /ISH–; cohort 2, IHC 1 +). The confirmed ORR was 26.3% in Cohort 1 and 9.5% in Cohort 2. The median OS was 7.8 months in cohort 1 and 8.5 months in cohort 2[133]. These results provide initial evidence that T-DXd has clinical benefits in patients with heavily pretreated HER2-low G/GEJ cancers.

Similarly, T-Dxd in the DESTINY-Gastric02 trial also achieved encouraging results in 2L western GC patients with a cORR of 41.8% and a median PFS of 5.6 months [134]. Other trials, such as phase III 2L DESTINY-Gastric04 and phase III 1L DESTINY-Gastric03, are also in progress (NCT04379596, NCT04704934).

**Disitamab vedotin (RC48)**

Disitamab vedotin (RC48) is a novel HER2-ADC drug independently developed in China, which is composed of three parts: anti-HER2 extracellular domain antibody, MC-Val-Cit-PAB linker, and cytotoxin monomethyl auristatin E (MMAE) [135]. This novel antibody has a stronger affinity to HER2 than the standard of care. Unlike T-DM1, disitamab vedotin has a bypass-killing effect on nearby tumor cells regardless of HER2 status, which could help overcome spatial heterogeneity and enhance anti-tumor effects. RC48 was well tolerated and showed promising antitumor activity in patients with HER2-positive advanced GC in a phase I trial [136]. The phase II RC48-C008 trial revealed a significant benefit of RC48 with HER2-overexpressing GC patients who had undergone at least two prior lines of therapy, in which the ORR was 24.8%, mPFS was 4.1 months and mOS was 7.9 months [137]. Of note, the ORR of RC48 in patients with HER2 IHC2 + /FISH- was 16.7%, slightly lower than in HER2-positive patients. These findings indicated that RC48 exerted considerable anti-tumor effectiveness and tolerable safety in patients with HER2-positive GC, as well as in those with HER2 low expression GC. In June 2021, disitamab vedotin was approved in China for the treatment of patients with HER2-overexpressing advanced or metastatic G/GEJ adenocarcinoma who received at least two systemic chemotherapy regimens. The ongoing phase III RC48-C007 (NCT04714190) trial aims to evaluate the efficacy and safety of RC48 as a third-line treatment and beyond in patients with advanced HER2-positive GC.

**Other ADCs**

ARX788 is another investigational anti-HER2 antibody–drug conjugate consisting of HER2-targeted monoclonal antibody (mAb) coupled with a highly effective tubulin inhibitor (AS269). ARX788 was well tolerated and had a promising anti-tumor effect in HER2-positive GC patients previously treated with trastuzumab-based regimens in a phase I multicenter dosage expansion trial [138]. The ORR was confirmed to be 37.9%, and the DCR was 55.2%. With a median follow-up period of 10 months, the mPFS and OS were 4.1 and 10.7 months, respectively. On March 18, 2021, the FDA granted ARX788 as an orphan drug for treating HER2-positive GC. A randomized controlled, multicenter, open-label phase II/III study is underway to assess the efficacy of ARX788 as second-line treatment for HER2-positive advanced G/GEJ adenocarcinoma (Chinadrugtrials.org.cn: CTR20211583).

##### Tyrosine kinase inhibitors

**Tucatinib**

Tucatinib, a highly selective HER2-directed tyrosine kinase inhibitor (TKI), was approved by FDA for HER2-positive metastatic breast cancer in 2020 and is under exploration in GC. In preclinical studies, tucatinib plus trastuzumab demonstrated superior activity compared to a single agent in GEC xenograft models [139]. Recently, the phase II/III MOUNTAINEER-02 (NCT04499924) was initiated to evaluate the efficacy of tucatinib, trastuzumab combined with ramucirumab, and paclitaxel in previously treated HER2 + advanced G/GEJ adenocarcinoma [140].

Other novel therapeutic approaches are being under investigation, including anti-HER2 CAR-T-cell therapy (NCT04511871, NCT04650451), CAR-natural killer cell (NK) therapy [141], and CAR-macrophage (CAR-M) therapy (NCT04660929), B-cell and monocyte-based immunotherapeutic vaccines (BVAC-B), BAY2701439 and CAM-H2 targeted HER2 radiotherapy (NCT04147819, NCT04467515). These widespread attempts at HER2-targeted CAR cell therapy in solid tumors may hopefully lead to the development of new drug candidates in patients with HER2-positive GC.

#### Antiangiogenic therapy

Blocking angiogenesis is a key strategy in GC therapy, including anti-VEGF monoclonal antibodies, VEGF-binding proteins, and VEGF receptor TKIs (Table 5) [142]. Ramucirumab, a typical antiangiogenic monoclonal antibody, targets VEGFR-2 and is approved by the FDA for treating advanced GC [143]. In the second-line REGARD trial, ramucirumab demonstrated significant improvement in patient OS and PFS versus best supportive care in metastatic GC [144]. In the RAINBOW trial, when coupled with paclitaxel, ramucirumab significantly prolonged overall survival compared to paclitaxel alone [145]. Similarly, results from RAINBOW-Asia bridging study also supported the application of ramucirumab plus paclitaxel as second-line therapy in a predominantly Chinese population with advanced gastric or GEJ adenocarcinoma [146]. However, neither ramucirumab nor bevacizumab brought extra survival benefits when added to platinum or fluoropyrimidine chemotherapy in GC patients in the first-line settings [147, 148].

**Table 5:** Summary of other important investigational targeted therapies for HER2-negative mGC

Target	Approach	Agent	Trial	Phase	Lines	Treatment	Outcomes	References
CLDN18.2	CAR T	CT041	Shen et al	I	2nd line and beyond	CLDN18.2-targeted CAR T cells (CT041)	ORR 57.1%; DCR 75.0%; 6-month OS rate 81.2%; mPFS 4.2 months (GC cohort)	[165]
VEGFR	TKI	Levantinib	EPOC1706	II	1st and 2nd-line	Levantinib + pembrolizumab	ORR 69%; mPFS 7.1 months	[151]
			LEAP-005	II	3rd line and beyond	Levantinib + pembrolizumab	ORR 10%; DCR 48%; mPFS 2.5 months; mOS 5.9 months (GC cohort)	[192]
		Regorafenib	REGONIVO	Ib	3rd line and beyond	Regorafenib + nivolumab	ORR 44%; mPFS 5.6 months	[150]
			INTEGRATE IIa	III	3rd line and beyond	Regorafenib vs. placebo	mOS 4.5 vs. 4.0 months; mPFS 1.8 vs. 1.6 months	[149]
		Fruquintinib	Zhang, Y., et al	Ib/II	2nd line	Fruquintinib + paclitaxel	mPFS 4 months; mOS 8.5 months; ORR 25.9%; DCR 66.7% (in the 4 mg dose cohort)	[154]
			FRUTIGA	III	2nd line	Fruquintinib + paclitaxel vs. placebo + paclitaxel	Ongoing	NCT03223376
FGFR	Monoclonal antibody	Bemarituzumab	FIGHT	II	1st line	Bemarituzumab + mFOLFOX6 vs placebo + mFOLFOX6	ORR 47% vs. 33%; mPFS 9.5 vs. 7.4 months	[172]
	TKI	Futibatinib	Meric-Bernstam, F., et al	I	3rd line and beyond	Futibatinib	ORR 22.2%; DCR 55.6% (GC cohort)	[169]

Regorafenib is an oral multi-kinase inhibitor targeting angiogenic, stromal and oncogenic receptor tyrosine kinases (RTK). Results from a phase III trial (INTEGRATE IIa) presented at ASCO GI 2023 demonstrated that regorafenib significantly improved OS (4.5 months vs. 4.0 months; HR = 0.52; *P* = 0.011) in patients with advanced gastro-oesophageal cancer (AGOC) in later-line settings [149]. Meanwhile, other studies exploring the efficacy of anti-VEGF and anti-PD1 combination in GC populations are also under investigation. The combination of regorafenib and nivolumab had a manageable safety profile and effective antitumor activity in a phase I trial for the GC subgroup [150]. INTEGRATE IIb ((NCT0487936)), an international randomized phase 3 trial, is ongoing to compare regorafenib plus nivolumab to standard chemotherapy in pre-treated patients with AGOC. Besides, lenvatinib plus pembrolizumab showed promising anti-tumor activity with an ORR of 69% in the first-line and second-line treatment of advanced GC [151].

Apatinib is a small molecule VEGFR inhibitor with China Food and Drug Administration (CFDA) approval for the treatment of advanced or metastatic chemotherapy-refractory GC. Apatinib improved median PFS and OS versus placebo in Chinese patients with advanced gastric or gastroesophageal junction adenocarcinoma in the third line and beyond[152]. Most of the patients in this trial did not receive prior antiangiogenic therapies since they were not standard treatments in China at that time, so clinical evidence is still lacking for the use of apatinib in patients who previously received ramucirumab. Unfortunately, no significant improvements were observed in overall survival (OS) in western populations in the phase III ANGEL clinical trial [153].

Fruquintinib is a highly selective VEGFR family kinase inhibitor that targets VEGFR1, 2 and 3 and is independently developed in China. Fruquintinib was approved in China by the NMPA in September 2018 and commercially launched in late November 2018 as a third-line treatment for patients with metastatic colorectal cancer. In a phase Ib/II study, adding fruquintinib to paclitaxel as second-line treatment for mGC patients at recommended phase 2 dose (RP2D) showed an mPFS of 4 months and mOS of 8.5 months. In the 4 mg dose cohort of 27 patients with evaluable tumor response, the ORR was 25.9% and the DCR was 66.7%[154]. A randomized phase III FRUTIGA study has investigated fruquintinib plus paclitaxel versus paclitaxel alone in patients with advanced gastric or gastroesophageal junction (GEJ) adenocarcinoma who had progressed after first-line standard chemotherapy (NCT03223376). Initial results from FRUTIGA showed that fruquintinib combined with paclitaxel showed significant improvements in PFS, ORR and DCR. Full detailed results are still being analyzed and will be revealed soon.

#### Other biomarker-targeted therapy

Novel diagnostic techniques have contributed to characterizing the genetic profile of GC and identifying new potential molecular targets. Recently, researchers have looked into Claudin-18.2-targeted therapy, fibroblast growth receptor (FGFR) pathway inhibitors, and EGFR inhibitors as effective targeted therapies to treat advanced GC (Table 5). Although emerging innovative drugs have made remarkable progress in GC treatments, extensive clinical explorations are needed to advance precision medicine.

##### CLAUDIN 18.2-targeted therapy

Claudin 18.2 (CLDN18.2), a component of intercellular junctions [155], is exclusively detected in gastric mucosa and absent from other healthy tissues. Upon malignant transformation, CLDN18.2 expression can be retained in various tumor tissues, including G/GEJ cancer and especially diffuse-type GC [156]. The prevalence of CLDN18.2 overexpression in GC varies wildly among studies ranging from 14.1% to 72% [157–159].

Zolbetuximab is a chimeric IgG1 monoclonal antibody that binds to CLDN18.2 and induces antibody-dependent and complement-dependent cytotoxicity [160]. To date, zolbetuximab has shown great potential to become a valuable target in GC. In the phase II MONO study, single-agent zolbetuximab achieved an ORR of 9% and a disease control rate of 23% in 43 patients with previously treated oesophageal or G/GEJ cancers [161]. A randomized phase II study (FAST) indicated that zolbetuximab plus first-line chemotherapy significantly improved PFS and OS in patients with CLDN18.2-positive G/GEJ cancer [159]. Subgroup analysis indicated a correlation between moderate-to-strong CLDN18.2 expression and a better overall survival rate. In the phase III SPOTLIGHT trial, zolbetuximab plus mFOLFOX6 significantly improved mPFS (10.61 vs 8.67 months, HR 0.751, *P* = 0.0066) and mOS (18.23 vs 15.54 months, HR 0.750, *P* = 0.0053) in patients with CLDN18.2-positive and HER-2-negative advanced G/GEJ cancer[162].

GLOW (NCT03653507) is another phase III trial investigating zolbetuximab plus CAPOX as first-line treatment in patients with CLDN18.2-positive, HER2-negative, locally advanced unresectable or metastatic gastric or GEJ cancer. In this study, zolbetuximab plus CAPOX showed a significant improvement in mPFS (8.21 vs 6.80 months, HR 0.687, *P* = 0.0007) and mOS (14.39 vs 12.16 months, HR 0.771, *P* = 0.0118) compared to placebo plus CAPOX[163]. Additionally, zolbetuximab is also being studied in combination with immunotherapy in patients with CLDN18.2-positive advanced gastric or GEJ cancer in the ILUSTRO study (NCT03505320).

Another promising therapeutic approach targeting CLDN18.2 employs CLDN18.2-specific chimeric antigen receptor (CAR) T cells. CLDN18.2-specific CAR T cells achieved partial or complete tumor regression in CLDN18.2-positive PDX models [164]. A phase I study of CLDN18.2-specific CAR T cells in gastrointestinal cancers conducted by Prof. Shen Lin's team demonstrated that in GC patients, the ORR and DCR were 57.1% and 75.0%, respectively, and the 6-month overall survival rate was 81.2% [165]. Claudin 18.2 served as a new target for the later-line treatment of GC, with considerable ORR improvement achieved in Claudin 18.2 CAR-T therapy, which has become a hallmark event for cellular immunotherapy in solid tumors. Currently, several new drugs focusing on Claudin 18.2, such as Claudin 18.2 bispecific antibodies (Claudin 18.2/CD3, Claudin 18.2/PD-L1) and ADC analogs, are being developed. Although these drugs have not been approved for clinical applications, some of them showed promising preclinical data and are being widely studied in different clinical trials. Since Claudin 18.2 is also expressed on the normal gastric mucosal epithelial surface, the risk of adverse reactions and whether ADC drugs may aggravate normal mucosal damage should also be a concern.

##### FGFR-targeted therapy

FGFR1 mutations, FGFR2 amplifications, and FGFR3 rearrangements are the most common FGFR alterations in GC [166]. Different types of FGFR targeting agents were explored or developed in GC, including multikinase inhibitors, pan-FGFR inhibitors, FGFR1-3 inhibitors, selective FGFR inhibitors and ADC. Nevertheless, most multikinase inhibitor studies were preclinical or single case reports in GC without robust clinical evidence [167]. Futibatinib, an irreversible and highly selective FGFR1–4 inhibitor that permanently disables FGFR2, has been tested in a phase II trial involving patients with advanced-stage solid tumors harboring FGFR alterations, including those with FGFR2-amplified G/GEJ cancers [168]. Although the ORR was reported to be 22.2% in the GC cohort [169], more data are needed to support the efficacy of multiple FGFR inhibitors in different FGFR gene alterations in GC.

Currently, bemarituzumab has shown some promising results in the treatment of mGC [170]. It is a first-in-class afucosylated monoclonal antibody against the FGFR2b splice variant frequently overexpressed in FGFR2- amplified G/GEJ cancers. In a phase I trial, 17.9% of patients with FGFR2 amplifications had a confirmed response to bemarituzumab [171]. Based on the safety and activity profile of bemarituzumab monotherapy in GC, the phase II FIGHT trial was designed to evaluate the efficacy of bemarituzumab plus mFOLFOX6 regimen in previously untreated, FGFR2b-overexpressing advanced-stage G/GEJ cancers [172]. The trial showed a 2-month improvement in PFS, and the OS was not reached (NR) in the experimental arm (bemarituzumab + mFOLFOX6). However, the experimental arm had a higher incidence of adverse events than the control chemotherapy arm, particularly in regard to ocular toxicity.

##### EGFR-targeted therapy

Approximately 5–10% of patients with G/GEJ cancers have EGFR amplifications or EGFR overexpression, both of which are associated with poor prognosis [173]. Previous large randomized clinical trials have failed to demonstrate any significant survival benefit with EGFR-targeted agents [92, 174], perhaps because most of the studies were performed in unselected patient populations regardless of EGFR status. Besides, biomarker analysis of the EXPAND and COG trials suggests activity in patients with tumors expressing high levels of EGFR, thus supporting the significance of patient selection for future trials [175, 176]. In a prospective cohort, patients with metastatic gastroesophageal adenocarcinoma were screened for EGFR amplification and subsequently treated with anti-EGFR therapy (cetuximab). The ORR was 58% (4 of 7 patients), and the DCR was 100% (7 of 7 patients), implying that EGFR inhibition should be further studied in selected patients [177]. Many of the ongoing EGFR inhibitor studies should test EGFR alterations in the GC patients prior to enrollment to overcome resistance to EGFR-targeted therapies.

##### MET/HGF pathway inhibitors

c-Mesenchymal-Epithelial Transition (c-MET) is a tyrosine kinase receptor from MET families, and hepatocyte growth factor (HGF) is the common ligand to c-MET [178]. MET/HGF pathway activation is associated with tumor invasiveness and poor disease prognosis. The anti-MET monoclonal antibody, onartuzumab, has been studied in a phase III trial of onartuzumab plus mFOLFOX6 vs placebo plus mFOLFOX6 in patients with metastatic HER2-negative G/GEJ cancers. However, the addition of onartuzumab to mFOLFOX6 did not improve clinical outcomes in the ITT population or in the MET-positive population [179]. Rilotumumab is a humanized monoclonal antibody targeting HGF. Two phase III trials (RILOMET-1 and RILOMET-2) investigated rilotumumab plus chemotherapy in advanced MET-positive G/GEJ cancers. Unfortunately, both studies were terminated due to increased number of deaths in the rilotumumab group[180, 181]. Additionally, several selective/non-selective c-MET TKIs, such as tinvatinib, AMG 337 and foretinib, have also been tested in MET-positive GC, but no significant benefit was seen in clinical trials[182–184].

## Challenges and future perspectives

Even though substantial advances have been made in the treatment of GC, further research and development are still necessary. Improving early detection, reducing recurrence and optimizing treatment strategies are the primary challenges and prospects for GC management. To increase GC early detection and promote patients’ overall survival, endoscopic screening programs should be implemented in high-risk regions, and more precise early detection technologies are of great value. In a previous study, we demonstrated an artificial intelligence (AI) diagnostic platform, GRAIDS, to detect upper gastrointestinal cancers using real-world endoscopic imaging data from six Chinese hospitals with varying experience in the endoscopic diagnosis of upper gastrointestinal cancer [185]. GRAIDS provided both real-time and retrospective assistance for enhancing the effectiveness of upper gastrointestinal cancer screening and diagnosis, with high diagnostic accuracy and sensitivity in detecting upper gastrointestinal cancers. In the near future, the AI system will help many physicians in community-based hospitals identify upper gastrointestinal cancers more efficiently and accurately [186].

In addition, recurrence of GC remains common despite the multimodality treatment, so many studies in progress aim to identify individuals at risk of recurrence after treatment. Circulating tumor DNA (ctDNA) can be detected in the circulation of cancer patients and has the potential to predict minimal residual disease [187]. Liquid biopsies can detect a broader spectrum of abnormalities in a heterogeneous tumor compared to conventional tissue biopsies. According to a study investigating perioperative therapies in patients in the CRITICS trial with resectable GC, the presence of ctDNA could predict recurrence when analyzed within nine weeks after preoperative treatment and after surgery in patients eligible for multimodal treatment [187]. These findings highlight the significance of ctDNA as a biomarker for predicting patient outcomes following perioperative cancer treatment and surgical resection in patients with GC. In another 1630-patient cohort of ctDNA results, genomic alterations were correlated with clinicopathologic characteristics and outcomes and provided prognostic and predictive information [188]. As for advanced GC, ctDNA also serves as a potential biomarker of immunotherapy response, and its potential role in predicting irAEs is worth further investigation [189]. Further research aimed at prospectively collecting ctDNA is needed to confirm these findings. The existence of persistent ctDNA following curative-intent treatment of GC may indicate minimal residual disease, and trials are underway to determine whether additional adjuvant therapy can result in the clearance of ctDNA.

Intratumoral, intrapatient, and interpatient heterogeneity in GC is the major barrier to drug development for systemic therapies. Most GC patients are not susceptible to immune checkpoint inhibitor monotherapies. Thus, one of the major challenges in systemic treatments for GC is overcoming resistance to ICI therapy. One strategy is to develop novel ICIs with better efficacy. Recently, many novel immune checkpoint modulators have been widely investigated, including LAG-3, VISTA, TIM-3, TIGIT, CD38, CD39, and CD73[190]. Another key strategy is combining ICI and other therapies, such as other ICI, targeted therapies, other immune-modulating agents, chemotherapy (as discussed above), and radiotherapy [191]. As mentioned above, in the CheckMate-649 study, the combination of anti-PD-1 and anti-CTLA-4 agents (nivolumab plus ipilimumab) failed to improve treatment outcomes compared to traditional chemotherapy [57]. In the EPOC1706 study, lenvatinib, an anti-angiogenic multiple receptor tyrosine kinase inhibitor, combined with pembrolizumab showed an exciting activity with an ORR of 69% in the first-line and second-line treatment of advanced GC[151]. ICI combined with other anti-immunosuppressive factor agents, such as anti-transforming growth factor-β (TGF-β), is also being investigated in clinical trials (NCT04856774). To fully understand the mechanism of resistance to immunotherapy, factors such as epigenetics, metabolism, immune suppression, and microbiota must be considered. Therefore, the development of combined therapies should be based on understanding the underlying mechanisms of immune modulation and resistance, rather than simply combining available therapies in a haphazard manner.

Rapid developments are ongoing in the clinical use of ADCs and are now considered one of the current hot spots for antitumor drug development. In particular, ADCs have emerged as a new era of targeted therapy in the field of GC treatment. The latest generation of ADCs has expanded the treatment population to include novel targets and demonstrated superior clinical outcomes compared to traditional chemotherapy drugs. Nevertheless, certain aspects of ADCs remain to be addressed. Firstly, it is necessary to explore ways to advance ADCs as first-line therapy to benefit a larger number of patients. Secondly, to make better use of medical resources, a more differentiated target layout needs to be established, moving beyond the focus on distinct targets such as HER2. To address these challenges, optimization of the toxin, linker and toxicity of ADCs is essential, along with the development of ADC-combination therapies to improve efficacy. We anticipate the discovery of more potential ADC drugs and expect a breakthrough in first-line treatment.

Currently, many clinical trials have complex treatment regimens, including mono-immunotherapy, double-checkpoint inhibitors, anti-angiogenic drugs, and biomarker-directed therapies [190, 192]. However, the challenge of determining the optimal treatment strategy and the appropriate timing of molecular biomarker screening has yet to be resolved. We expect that extensive translational research, preclinical investigations, and multi-omics-based clinical trials will lead to breakthroughs in the diagnosis and treatment of GC. Therefore, we eagerly anticipate future studies that have the potential to improve clinical practice in the coming years.

## Data Availability

Not applicable.

## Abbreviations

ADC: Antibody–drug conjugates
ADCC: Antibody-dependent cell-mediated cytotoxicity
AGOC: Advanced gastro-oesophageal cancer
AI: Artificial intelligence
ASCO: American Society of Clinical Oncology
BsAbs: Bispecific antibodies
BSC: Best supportive care
CAPOX: Capecitabine and oxaliplatin
CAR: Chimeric antigen receptor
CCNE1: Cyclin E1
CF: Cisplatin and fluorouracil
CFDA: China Food and Drug Administration
CI: Confidence interval
CLDN18.2: Claudin18.2
CPS: Combined positive score
CRT: Chemoradiotherapy
CSCO: Chinese Society for Clinical Oncology
ctDNA: Circulating tumor DNA
CTLA-4: Cytotoxic T lymphocyte antigen-4
DCR: Disease control rate
DFS: Disease-free survival
dMMR: Mismatch-repair deficiency
DoR: Duration of response
DOS: Docetaxel, oxaliplatin, and S-1
EBV: Epstein-Barr virus
EBVaGC: Epstein-Barr virus-associated gastric cancer
ECD: Extracellular domain
ECF: Epirubicin, cisplatin, and fluorouracil
EFS: Event-free survival
EGFR: Epidermal growth factor receptor
EOX: Epirubicin and oxaliplatin
ERBB: Erythroblastic leukemia viral oncogene homolog
ERK: Extracellular regulated protein kinase
ESMO: European Society for Medical Oncology
FDA: Food and Drug Administration
FGFR: Fibroblast growth factor receptor
FISH: Fluorescent in situ hybridization
FLOT: Fluorouracil, leucovorin, oxaliplatin, and docetaxel
FOLFOX: Fluorouracil, leucovorin, and oxaliplatin
FP: Fluorouracil and cisplatin
GC: Gastric cancer
GEA: Gastroesophageal junction adenocarcinoma
GEJ: Gastroesophageal junction
HER2: Human epidermal growth factor receptor 2
HR: Hazard ratio
ICI: Immune checkpoint inhibitor
IHC: Immunohistochemistry
KRAS: Kirsten rats sarcomaviral oncogene homolog
LAG-3: Lymphocyte-activation gene 3
mAbs: Monoclonal antibodies
MAPK: Mitogen-activated protein kinase
MET: Mesenchymal epithelial transition
MMAE: Cytotoxin monomethyl auristatin E
MSI: Microsatellite instability
mTOR: Mammalian target of rapamycin
NCCN: National Comprehensive Cancer Network
NE: Not evaluable
NK: Natural killer
ORR: Objective response rate
OS: Overall survival
pCR: Pathological complete response
PD-1: Programmed cell death 1
PD-L1: Programmed cell death ligand 1
PFS: Progression-free survival
PI3K: Phosphatidylinositol-3-kinase
PTEN: Phosphatase and tensin homolog
RFS: Relapse-free survival
RP2D: Recommended phase 2 dose
RTK: Receptor tyrosine kinase
SOX: S-1 and oxaliplatin
SOXRT: S-1, oxaliplatin and radiotherapy
SP: S-1 and cisplatin
TAP: Tumor area positivity
TCGA: The Cancer Genome Atlas
T-DM1: Trastuzumab emtansine
T-DXd: Trastuzumab deruxtecan
TGF-β: Transforming growth factor-β
TIGIT: T cell immunoreceptor with Ig and ITIM domain
TIM-3: T cell immunoglobulin and mucin domain 3
TKIs: Tyrosine kinase inhibitors
TMB: Tumor mutational burden
VEGF: Vascular endothelial growth factor
VISTA: V-domain Ig suppressor of T cell activation
XP: Capecitabine and cisplatin

## References

[1] Siegel RL (2021). Cancer statistics, 2021. CA Cancer J Clin.

[2] Sung H (2021). Global Cancer Statistics 2020: GLOBOCAN estimates of incidence and mortality worldwide for 36 cancers in 185 countries. CA Cancer J Clin.

[3] Tan P, Yeoh KG (2015). Genetics and molecular pathogenesis of gastric adenocarcinoma. Gastroenterology.

[4] Tramacere I (2012). A meta-analysis on alcohol drinking and gastric cancer risk. Ann Oncol.

[5] Lordick F (2022). Gastric cancer: ESMO clinical practice guideline for diagnosis, treatment and follow-up. Ann Oncol.

[6] Lu L (2021). A global assessment of recent trends in gastrointestinal cancer and lifestyle-associated risk factors. Cancer Commun (Lond).

[7] Pennathur A (2013). Oesophageal carcinoma. Lancet.

[8] Qiu H, Cao S, Xu R (2021). Cancer incidence, mortality, and burden in China: a time-trend analysis and comparison with the United States and United Kingdom based on the global epidemiological data released in 2020. Cancer Commun (Lond).

[9] Wagner AD (2017). Chemotherapy for advanced gastric cancer. Cochrane Database Syst Rev.

[10] Korfer J, Lordick F, Hacker UT. Molecular targets for gastric cancer treatment and future perspectives from a clinical and translational point of view. Cancers (Basel), 2021;13(20). 10.3390/cancers13205216 PMC8533881 34680363

[11] Cancer Genome Atlas Research N. Comprehensive molecular characterization of gastric adenocarcinoma. Nature, 2014;513(7517): 202–9. 10.1038/nature13480 PMC4170219 25079317

[12] Salem ME (2018). Comparative molecular analyses of esophageal squamous cell carcinoma, esophageal adenocarcinoma, and gastric adenocarcinoma. Oncologist.

[13] Wang J (2021). Large-scale analysis of KMT2 mutations defines a distinctive molecular subset with treatment implication in gastric cancer. Oncogene.

[14] Bang YJ (2010). Trastuzumab in combination with chemotherapy versus chemotherapy alone for treatment of HER2-positive advanced gastric or gastro-oesophageal junction cancer (ToGA): a phase 3, open-label, randomised controlled trial. Lancet.

[15] Chao J (2021). Assessment of pembrolizumab therapy for the treatment of microsatellite instability-high gastric or gastroesophageal junction cancer among patients in the KEYNOTE-059, KEYNOTE-061, and KEYNOTE-062 Clinical Trials. JAMA Oncol.

[16] Nakamura Y (2021). Biomarker-targeted therapies for advanced-stage gastric and gastro-oesophageal junction cancers: an emerging paradigm. Nat Rev Clin Oncol.

[17] Wang FH (2021). The Chinese Society of Clinical Oncology (CSCO): Clinical guidelines for the diagnosis and treatment of gastric cancer, 2021. Cancer Commun (Lond).

[18] Ajani JA (2022). Gastric cancer, version 2, 2022, NCCN clinical practice guidelines in oncology. J Natl Compr Cancer Netw.

[19] Cunningham D (2006). Perioperative chemotherapy versus surgery alone for resectable gastroesophageal cancer. N Engl J Med.

[20] Ychou M (2011). Perioperative chemotherapy compared with surgery alone for resectable gastroesophageal adenocarcinoma: an FNCLCC and FFCD multicenter phase III trial. J Clin Oncol.

[21] Al-Batran SE (2019). Perioperative chemotherapy with fluorouracil plus leucovorin, oxaliplatin, and docetaxel versus fluorouracil or capecitabine plus cisplatin and epirubicin for locally advanced, resectable gastric or gastro-oesophageal junction adenocarcinoma (FLOT4): a randomised, phase 2/3 trial. Lancet.

[22] Kang YK (2021). PRODIGY: a phase III study of neoadjuvant docetaxel, oxaliplatin, and S-1 plus surgery and adjuvant S-1 versus surgery and adjuvant S-1 for resectable advanced gastric cancer. J Clin Oncol.

[23] Yoshida K (2019). Addition of docetaxel to oral fluoropyrimidine improves efficacy in patients with stage III gastric cancer: interim analysis of JACCRO GC-07, a randomized controlled trial. J Clin Oncol.

[24] Zhang X (2021). Perioperative or postoperative adjuvant oxaliplatin with S-1 versus adjuvant oxaliplatin with capecitabine in patients with locally advanced gastric or gastro-oesophageal junction adenocarcinoma undergoing D2 gastrectomy (RESOLVE): an open-label, superiority and non-inferiority, phase 3 randomised controlled trial. Lancet Oncol.

[25] Japanese Gastric Cancer, A.Japanese Gastric Cancer Treatment Guidelines 2021 (6th edition). Gastric Cancer, 2023;26(1): 1–25. 10.1007/s10120-022-01331-8 PMC9813208 36342574

[26] Bang YJ (2012). Adjuvant capecitabine and oxaliplatin for gastric cancer after D2 gastrectomy (CLASSIC): a phase 3 open-label, randomised controlled trial. Lancet.

[27] Noh SH (2014). Adjuvant capecitabine plus oxaliplatin for gastric cancer after D2 gastrectomy (CLASSIC): 5-year follow-up of an open-label, randomised phase 3 trial. Lancet Oncol.

[28] Sasako M (2011). Five-year outcomes of a randomized phase III trial comparing adjuvant chemotherapy with S-1 versus surgery alone in stage II or III gastric cancer. J Clin Oncol.

[29] Pietrantonio F (2019). Individual patient data meta-analysis of the value of microsatellite instability as a biomarker in gastric cancer. J Clin Oncol.

[30] Macdonald JS (2001). Chemoradiotherapy after surgery compared with surgery alone for adenocarcinoma of the stomach or gastroesophageal junction. N Engl J Med.

[31] Lee J (2012). Phase III trial comparing capecitabine plus cisplatin versus capecitabine plus cisplatin with concurrent capecitabine radiotherapy in completely resected gastric cancer with D2 lymph node dissection: the ARTIST trial. J Clin Oncol.

[32] Park SH (2021). A randomized phase III trial comparing adjuvant single-agent S1, S-1 with oxaliplatin, and postoperative chemoradiation with S-1 and oxaliplatin in patients with node-positive gastric cancer after D2 resection: the ARTIST 2 trial. Ann Oncol.

[33] Hofheinz RD (2021). Trastuzumab in combination with 5-fluorouracil, leucovorin, oxaliplatin and docetaxel as perioperative treatment for patients with human epidermal growth factor receptor 2-positive locally advanced esophagogastric adenocarcinoma: A phase II trial of the Arbeitsgemeinschaft Internistische Onkologie Gastric Cancer Study Group. Int J Cancer.

[34] Hofheinz RD (2020). Perioperative trastuzumab and pertuzumab in combination with FLOT versus FLOT alone for HER2-positive resectable esophagogastric adenocarcinoma: final results of the PETRARCA multicenter randomized phase II trial of the AIO. J Clin Oncol.

[35] Rivera F (2021). Perioperative trastuzumab, capecitabine and oxaliplatin in patients with HER2-positive resectable gastric or gastro-oesophageal junction adenocarcinoma: NEOHX phase II trial. Eur J Cancer.

[36] Wagner AD (2019). EORTC-1203-GITCG - the "INNOVATION"-trial: Effect of chemotherapy alone versus chemotherapy plus trastuzumab, versus chemotherapy plus trastuzumab plus pertuzumab, in the perioperative treatment of HER2 positive, gastric and gastroesophageal junction adenocarcinoma on pathologic response rate: a randomized phase II-intergroup trial of the EORTC-Gastrointestinal Tract Cancer Group, Korean Cancer Study Group and Dutch Upper GI-Cancer group. BMC Cancer.

[37] Cunningham D (2017). Peri-operative chemotherapy with or without bevacizumab in operable oesophagogastric adenocarcinoma (UK Medical Research Council ST03): primary analysis results of a multicentre, open-label, randomised phase 2–3 trial. Lancet Oncol.

[38] Goetze TO (2022). Perioperative ramucirumab in combination with FLOT versus FLOT alone for resectable esophagogastric adenocarcinoma (RAMSES/FLOT7) with high rate of signet cell component: final results of the multicenter, randomized phase II/III trial of the German AIO and Italian GOIM. J Clin Oncol.

[39] Al-Batran S-E (2022). Surgical and pathological outcome, and pathological regression, in patients receiving perioperative atezolizumab in combination with FLOT chemotherapy versus FLOT alone for resectable esophagogastric adenocarcinoma: Interim results from DANTE, a randomized, multicenter, phase IIb trial of the FLOT-AIO German Gastric Cancer Group and Swiss SAKK. J Clin Oncol.

[40] Liu Y (2021). Camrelizumab combined with FLOFOX as neoadjuvant therapy for resectable locally advanced gastric and gastroesophageal junction adenocarcinoma: updated results of efficacy and safety. J Clin Oncol.

[41] Li H (2021). Phase II study of perioperative toripalimab in combination with FLOT in patients with locally advanced resectable gastric/gastroesophageal junction (GEJ) adenocarcinoma. J Clin Oncol.

[42] Alcindor T (2021). Phase II trial of perioperative chemotherapy + avelumab in locally advanced gastroesophageal adenocarcinoma: preliminary results. J Clin Oncol.

[43] Li S (2021). A prospective, phase II, single-arm study of neoadjuvant/conversion therapy with camrelizumab, apatinib, S-1 ± oxaliplatin for locally advanced cT4a/bN+ gastric cancer. J Clin Oncol.

[44] Wei J (2021). SHARED: Efficacy and safety of sintilimab in combination with concurrent chemoradiotherapy (cCRT) in patients with locally advanced gastric (G) or gastroesophageal junction (GEJ) adenocarcinoma. J Clin Oncol.

[45] Bang YJ (2019). KEYNOTE-585: Phase III study of perioperative chemotherapy with or without pembrolizumab for gastric cancer. Future Oncol.

[46] Janjigian YY, et al. MATTERHORN: efficacy and safety of neoadjuvant-adjuvant durvalumab and FLOT chemotherapy in resectable gastric and gastroesophageal junction cancer—a randomized, double-blind, placebo-controlled, phase 3 study. J Clin Oncol. 2021;39(15):TPS4151

[47] Andre T (2023). neoadjuvant nivolumab plus ipilimumab and adjuvant nivolumab in localized deficient mismatch repair/microsatellite instability-high gastric or esophagogastric junction adenocarcinoma: the GERCOR NEONIPIGA Phase II Study. J Clin Oncol.

[48] Pietrantonio F (2023). INFINITY: A multicentre, single-arm, multi-cohort, phase II trial of tremelimumab and durvalumab as neoadjuvant treatment of patients with microsatellite instability-high (MSI) resectable gastric or gastroesophageal junction adenocarcinoma (GAC/GEJAC). J Clin Oncol.

[49] Xu R-H (2019). S-1 plus oxaliplatin versus S-1 plus cisplatin as first-line treatment for advanced diffuse-type or mixed-type gastric/gastroesophageal junction adenocarcinoma: a randomized, phase 3 trial. J Clin Oncol.

[50] Hall PS (2021). efficacy of reduced-intensity chemotherapy with oxaliplatin and capecitabine on quality of life and cancer control among older and frail patients with advanced gastroesophageal cancer: the go2 phase 3 randomized clinical trial. JAMA Oncol.

[51] Shitara K (2017). Nab-paclitaxel versus solvent-based paclitaxel in patients with previously treated advanced gastric cancer (ABSOLUTE): an open-label, randomised, non-inferiority, phase 3 trial. Lancet Gastroenterol Hepatol.

[52] Shitara K (2018). Trifluridine/tipiracil versus placebo in patients with heavily pretreated metastatic gastric cancer (TAGS): a randomised, double-blind, placebo-controlled, phase 3 trial. Lancet Oncol.

[53] Shitara K (2020). Efficacy and safety of pembrolizumab or pembrolizumab plus chemotherapy vs chemotherapy alone for patients with first-line, advanced gastric cancer: the KEYNOTE-062 phase 3 randomized clinical trial. JAMA Oncol.

[54] Wainberg ZA (2022). Pembrolizumab with or without chemotherapy versus chemotherapy alone for patients with PD-L1–positive advanced gastric or gastroesophageal junction adenocarcinoma: Update from the phase 3 KEYNOTE-062 trial. J Clin Oncol.

[55] Rha SY (2023). VP1-2023: Pembrolizumab (pembro) plus chemotherapy (chemo) as first-line therapy for advanced HER2-negative gastric or gastroesophageal junction (G/GEJ) cancer: Phase III KEYNOTE-859 study. Ann Oncol.

[56] Janjigian YY (2021). First-line nivolumab plus chemotherapy versus chemotherapy alone for advanced gastric, gastro-oesophageal junction, and oesophageal adenocarcinoma (CheckMate 649): a randomised, open-label, phase 3 trial. Lancet.

[57] Janjigian YY (2021). LBA7 Nivolumab (NIVO) plus chemotherapy (Chemo) or ipilimumab (IPI) vs chemo as first-line (1L) treatment for advanced gastric cancer/gastroesophageal junction cancer/esophageal adenocarcinoma (GC/GEJC/EAC): CheckMate 649 study. Ann Oncol.

[58] Kang YK (2022). Nivolumab plus chemotherapy versus placebo plus chemotherapy in patients with HER2-negative, untreated, unresectable advanced or recurrent gastric or gastro-oesophageal junction cancer (ATTRACTION-4): a randomised, multicentre, double-blind, placebo-controlled, phase 3 trial. Lancet Oncol.

[59] Xu J (2021). LBA53 Sintilimab plus chemotherapy (chemo) versus chemo as first-line treatment for advanced gastric or gastroesophageal junction (G/GEJ) adenocarcinoma (ORIENT-16): first results of a randomized, double-blind, phase III study. Ann Oncol.

[60] Xu R-h, et al. Tislelizumab plus chemotherapy versus placebo plus chemotherapy as first-line therapy in patients with locally advanced unresectable or metastatic gastric or gastroesophageal junction (G/GEJ) adenocarcinoma. J Clin Oncol 2020;38(4_suppl): TPS458

[61] Moehler MH (2023). Rationale 305: Phase 3 study of tislelizumab plus chemotherapy vs placebo plus chemotherapy as first-line treatment (1L) of advanced gastric or gastroesophageal junction adenocarcinoma (GC/GEJC). J Clin Oncol.

[62] Moehler M (2021). Phase III trial of avelumab maintenance after first-line induction chemotherapy versus continuation of chemotherapy in patients with gastric cancers: results from JAVELIN gastric 100. J Clin Oncol.

[63] Fuchs CS (2018). Safety and efficacy of pembrolizumab monotherapy in patients with previously treated advanced gastric and gastroesophageal junction cancer: phase 2 clinical KEYNOTE-059 trial. JAMA Oncol.

[64] Kang YK (2017). Nivolumab in patients with advanced gastric or gastro-oesophageal junction cancer refractory to, or intolerant of, at least two previous chemotherapy regimens (ONO-4538-12, ATTRACTION-2): a randomised, double-blind, placebo-controlled, phase 3 trial. Lancet.

[65] Bang YJ (2018). Phase III, randomised trial of avelumab versus physician's choice of chemotherapy as third-line treatment of patients with advanced gastric or gastro-oesophageal junction cancer: primary analysis of JAVELIN Gastric 300. Ann Oncol.

[66] Triulzi T (2019). HER2 signaling regulates the tumor immune microenvironment and trastuzumab efficacy. Oncoimmunology.

[67] Varadan V (2016). Immune signatures following single dose trastuzumab predict pathologic response to preoperativetrastuzumab and chemotherapy in HER2-positive early breast cancer. Clin Cancer Res.

[68] Chaganty BKR (2018). Trastuzumab upregulates PD-L1 as a potential mechanism of trastuzumab resistance through engagement of immune effector cells and stimulation of IFNgamma secretion. Cancer Lett.

[69] Stagg J (2011). Anti-ErbB-2 mAb therapy requires type I and II interferons and synergizes with anti-PD-1 or anti-CD137 mAb therapy. Proc Natl Acad Sci U S A.

[70] Takahari D, et al. A phase Ib study of nivolumab plus trastuzumab with S-1/capecitabine plus oxaliplatin for HER2-positive advanced gastric cancer (Ni-HIGH study): safety evaluation. J Clin Oncol. 2020;38(15_suppl): 4525

[71] Rha SY, et al. A multi-institutional phase Ib/II trial of first-line triplet regimen (Pembrolizumab, Trastuzumab, Chemotherapy) for HER2-positive advanced gastric and gastroesophageal junction cancer (PANTHERA Trial): Molecular profiling and clinical update. J Clin. Oncol.2021;39(3_suppl):218.

[72] Janjigian YY (2020). First-line pembrolizumab and trastuzumab in HER2-positive oesophageal, gastric, or gastro-oesophageal junction cancer: an open-label, single-arm, phase 2 trial. Lancet Oncol.

[73] Janjigian YY (2021). The KEYNOTE-811 trial of dual PD-1 and HER2 blockade in HER2-positive gastric cancer. Nature.

[74] Guan WL (2021). The impact of mismatch repair status on prognosis of patients with gastric cancer: a multicenter analysis. Front Oncol.

[75] Vanderwalde A (2018). Microsatellite instability status determined by next-generation sequencing and compared with PD-L1 and tumor mutational burden in 11,348 patients. Cancer Med.

[76] Pietrantonio F (2021). Predictive role of microsatellite instability for PD-1 blockade in patients with advanced gastric cancer: a meta-analysis of randomized clinical trials. ESMO Open.

[77] Huang SC (2019). Subtraction of Epstein-Barr virus and microsatellite instability genotypes from the Lauren histotypes: combined molecular and histologic subtyping with clinicopathological and prognostic significance validated in a cohort of 1,248 cases. Int J Cancer.

[78] Qiu MZ (2020). Prospective observation: clinical utility of plasma Epstein-Barr virus DNA load in EBV-associated gastric carcinoma patients. Int J Cancer.

[79] Derks S (2016). Abundant PD-L1 expression in Epstein-Barr Virus-infected gastric cancers. Oncotarget.

[80] Kim ST (2018). Comprehensive molecular characterization of clinical responses to PD-1 inhibition in metastatic gastric cancer. Nat Med.

[81] Sun YT (2021). PD-1 antibody camrelizumab for Epstein-Barr virus-positive metastatic gastric cancer: a single-arm, open-label, phase 2 trial. Am J Cancer Res.

[82] Xie T (2020). Positive status of Epstein-Barr virus as a biomarker for gastric cancer immunotherapy: a prospective observational study. J Immunother.

[83] Mishima S (2019). Clinicopathological and molecular features of responders to nivolumab for patients with advanced gastric cancer. J Immunother Cancer.

[84] Shitara K (2018). Pembrolizumab versus paclitaxel for previously treated, advanced gastric or gastro-oesophageal junction cancer (KEYNOTE-061): a randomised, open-label, controlled, phase 3 trial. Lancet.

[85] Wainberg ZA (2021). Efficacy of pembrolizumab monotherapy for advanced gastric/gastroesophageal junction cancer with programmed death ligand 1 combined positive score >/=10. Clin Cancer Res.

[86] Moehler MH, et al. First-line (1L) nivolumab (NIVO) plus chemotherapy (chemo) versus chemo in advanced gastric cancer/gastroesophageal junction cancer/esophageal adenocarcinoma (GC/GEJC/EAC): expanded efficacy and safety data from CheckMate 649. J Clin Oncol. 2021;39(15_suppl): 4002

[87] Zhao JJ (2022). Low programmed death-ligand 1-expressing subgroup outcomes of first-line immune checkpoint inhibitors in gastric or esophageal adenocarcinoma. J Clin Oncol.

[88] Wang F (2019). Safety, efficacy and tumor mutational burden as a biomarker of overall survival benefit in chemo-refractory gastric cancer treated with toripalimab, a PD-1 antibody in phase Ib/II clinical trial NCT02915432. Ann Oncol.

[89] Shitara K, et al. The association of tissue tumor mutational burden (tTMB) using the Foundation Medicine genomic platform with efficacy of pembrolizumab versus paclitaxel in patients (pts) with gastric cancer (GC) from KEYNOTE-061. J Clin Oncol. 2020;38(15_suppl): 4537

[90] Marabelle A (2020). Association of tumour mutational burden with outcomes in patients with advanced solid tumours treated with pembrolizumab: prospective biomarker analysis of the multicohort, open-label, phase 2 KEYNOTE-158 study. Lancet Oncol.

[91] Yarden Y, Sliwkowski MX (2001). Untangling the ErbB signalling network. Nat Rev Mol Cell Biol.

[92] Abrahao-Machado LF, Scapulatempo-Neto C (2016). HER2 testing in gastric cancer: an update. World J Gastroenterol.

[93] Van Cutsem E (2015). HER2 screening data from ToGA: targeting HER2 in gastric and gastroesophageal junction cancer. Gastric Cancer.

[94] Qin S (2021). Treatment patterns and outcomes in chinese patients with gastric cancer by HER2 status: a noninterventional registry study (EVIDENCE). Oncologist.

[95] Tabernero J (2018). Pertuzumab plus trastuzumab and chemotherapy for HER2-positive metastatic gastric or gastro-oesophageal junction cancer (JACOB): final analysis of a double-blind, randomised, placebo-controlled phase 3 study. Lancet Oncol.

[96] Liu T (2019). Pertuzumab in combination with trastuzumab and chemotherapy for Chinese patients with HER2-positive metastatic gastric or gastroesophageal junction cancer: a subpopulation analysis of the JACOB trial. Cancer Commun (Lond).

[97] Thuss-Patience PC (2017). Trastuzumab emtansine versus taxane use for previously treated HER2-positive locally advanced or metastatic gastric or gastro-oesophageal junction adenocarcinoma (GATSBY): an international randomised, open-label, adaptive, phase 2/3 study. Lancet Oncol.

[98] Hecht JR (2016). Lapatinib in combination with capecitabine plus oxaliplatin in human epidermal growth factor receptor 2-positive advanced or metastatic gastric, esophageal, or gastroesophageal adenocarcinoma: TRIO-013/LOGiC–a randomized phase III trial. J Clin Oncol.

[99] Satoh T (2014). Lapatinib plus paclitaxel versus paclitaxel alone in the second-line treatment of HER2-amplified advanced gastric cancer in Asian populations: TyTAN–a randomized, phase III study. J Clin Oncol.

[100] Makiyama A (2020). Randomized, phase II study of trastuzumab beyond progression in patients with HER2-positive advanced gastric or gastroesophageal junction cancer: WJOG7112G (T-ACT study). J Clin Oncol.

[101] Yagi S (2019). Clinical significance of intratumoral HER2 heterogeneity on trastuzumab efficacy using endoscopic biopsy specimens in patients with advanced HER2 positive gastric cancer. Gastric Cancer.

[102] Nishida Y (2015). A novel gene-protein assay for evaluating HER2 status in gastric cancer: simultaneous analyses of HER2 protein overexpression and gene amplification reveal intratumoral heterogeneity. Gastric Cancer.

[103] Lee HJ (2015). Clinicopathologic significance of the intratumoral heterogeneity of HER2 gene amplification in HER2-positive breast cancer patients treated with adjuvant trastuzumab. Am J Clin Pathol.

[104] Kim KC (2011). Evaluation of HER2 protein expression in gastric carcinomas: comparative analysis of 1,414 cases of whole-tissue sections and 595 cases of tissue microarrays. Ann Surg Oncol.

[105] Haffner I (2021). HER2 expression, test deviations, and their impact on survival in metastatic gastric cancer: results from the prospective multicenter VARIANZ study. J Clin Oncol.

[106] Palle J (2020). Human epidermal growth factor receptor 2 (HER2) in advanced gastric cancer: current knowledge and future perspectives. Drugs.

[107] Park SR (2016). Extra-gain of HER2-positive cases through HER2 reassessment in primary and metastatic sites in advanced gastric cancer with initially HER2-negative primary tumours: results of GASTric cancer HER2 reassessment study 1 (GASTHER1). Eur J Cancer.

[108] Seo S (2019). Loss of HER2 positivity after anti-HER2 chemotherapy in HER2-positive gastric cancer patients: results of the GASTric cancer HER2 reassessment study 3 (GASTHER3). Gastric Cancer.

[109] Janjigian YY (2018). Genetic predictors of response to systemic therapy in esophagogastric cancer. Cancer Discov.

[110] Shen L (2018). Liquid biopsy: a powerful tool to monitor trastuzumab resistance in HER2-positive metastatic gastric cancer. Cancer Commun (Lond).

[111] Kwak EL (2015). Molecular heterogeneity and receptor coamplification drive resistance to targeted therapy in MET-amplified esophagogastric cancer. Cancer Discov.

[112] Kim J (2014). Preexisting oncogenic events impact trastuzumab sensitivity in ERBB2-amplified gastroesophageal adenocarcinoma. J Clin Invest.

[113] Kim ST (2018). Impact of genomic alterations on lapatinib treatment outcome and cell-free genomic landscape during HER2 therapy in HER2+ gastric cancer patients. Ann Oncol.

[114] Lee JY (2015). The impact of concomitant genomic alterations on treatment outcome for trastuzumab therapy in HER2-positive gastric cancer. Sci Rep.

[115] Sanchez-Vega F (2019). EGFR and MET amplifications determine response to HER2 Inhibition in ERBB2-amplified esophagogastric cancer. Cancer Discov.

[116] Wang DS (2019). Liquid biopsies to track trastuzumab resistance in metastatic HER2-positive gastric cancer. Gut.

[117] Sampera A (2019). HER-family ligands promote acquired resistance to trastuzumab in gastric cancer. Mol Cancer Ther.

[118] Gambardella V (2019). NRF2 through RPS6 activation is related to anti-HER2 drug resistance in HER2-amplified gastric cancer. Clin Cancer Res.

[119] Nordstrom JL (2011). Anti-tumor activity and toxicokinetics analysis of MGAH22, an anti-HER2 monoclonal antibody with enhanced Fcgamma receptor binding properties. Breast Cancer Res.

[120] Shinde A (2019). Can immunotherapy replace radiotherapy in melanoma brain metastases?. J Clin Oncol.

[121] Bang YJ (2017). First-in-human phase 1 study of margetuximab (MGAH22), an Fc-modified chimeric monoclonal antibody, in patients with HER2-positive advanced solid tumors. Ann Oncol.

[122] Catenacci DVT (2020). Margetuximab plus pembrolizumab in patients with previously treated, HER2-positive gastro-oesophageal adenocarcinoma (CP-MGAH22-05): a single-arm, phase 1b–2 trial. Lancet Oncol.

[123] Catenacci DVT, et al. Margetuximab with retifanlimab as first-line therapy in HER2+/PD-L1+ unresectable or metastatic gastroesophageal adenocarcinoma: MAHOGANY cohort A. ESMO Open. 2022;7(5) 10.1016/j.esmoop.2022.100563 PMC9588876 36029651

[124] Ku G (2021). 1380P Phase (Ph) II study of zanidatamab + chemotherapy (chemo) in first-line (1L) HER2 expressing gastroesophageal adenocarcinoma (GEA). Ann Oncol.

[125] Tabernero J (2022). HERIZON-GEA-01: Zanidatamab + chemo +/- tislelizumab for 1L treatment of HER2-positive gastroesophageal adenocarcinoma. Future Oncol.

[126] Zhang J (2022). First-in-human HER2-targeted bispecific antibody KN026 for the treatment of patients with HER2-positive metastatic breast cancer: results from a phase I Study. Clin Cancer Res.

[127] Xu J (2022). A phase II study evaluating KN026 monotherapy in patients (pts) with previously treated, advanced HER2-expressing gastric or gastroesophageal junction cancers (GC/GEJC). J Clin Oncol.

[128] Shen L (2022). 1210P The preliminary efficacy and safety of KN026 combined with KN046 treatment in HER2-positive locally advanced unresectable or metastatic gastric/gastroesophageal junction cancer without prior systemic treatment in a phase II study. Ann Oncol.

[129] Piha-Paul S (2020). O82 A phase 1 dose escalation study of PRS-343, a HER2/4–1BB bispecific molecule, in patients with HER2-positive malignancies. J Immunother Cancer.

[130] Criscitiello C, Morganti S, Curigliano G (2021). Antibody-drug conjugates in solid tumors: a look into novel targets. J Hematol Oncol.

[131] Ogitani Y (2016). Bystander killing effect of DS-8201a, a novel anti-human epidermal growth factor receptor 2 antibody-drug conjugate, in tumors with human epidermal growth factor receptor 2 heterogeneity. Cancer Sci.

[132] Shitara K (2020). Trastuzumab deruxtecan in previously treated HER2-positive gastric cancer. N Engl J Med.

[133] Yamaguchi K (2023). Trastuzumab deruxtecan in anti-human epidermal growth factor receptor 2 treatment-naive patients with human epidermal growth factor receptor 2–low gastric or gastroesophageal junction adenocarcinoma: exploratory cohort results in a phase II Trial. J Clin Oncol.

[134] Ku GY (2022). 1205MO Updated analysis of DESTINY-Gastric02: A phase II single-arm trial of trastuzumab deruxtecan (T-DXd) in western patients (Pts) with HER2-positive (HER2+) unresectable/metastatic gastric/gastroesophageal junction (GEJ) cancer who progressed on or after trastuzumab-containing regimen. Ann Oncol.

[135] Dai L (2022). Efficacy of disitamab vedotin in treating HER2 2+/FISH- gastric cancer. Onco Targets Ther.

[136] Xu Y (2021). Phase I study of the recombinant humanized anti-HER2 monoclonal antibody-MMAE conjugate RC48-ADC in patients with HER2-positive advanced solid tumors. Gastric Cancer.

[137] Peng Z (2021). Efficacy and safety of a novel anti-HER2 therapeutic antibody RC48 in patients with HER2-overexpressing, locally advanced or metastatic gastric or gastroesophageal junction cancer: a single-arm phase II study. Cancer Commun (Lond).

[138] Zhang Y (2022). Phase 1 multicenter, dose-expansion study of ARX788 as monotherapy in HER2-positive advanced gastric and gastroesophageal junction adenocarcinoma. Cell Rep Med.

[139] Kulukian A (2020). Preclinical activity of HER2-selective tyrosine kinase inhibitor tucatinib as a single agent or in combination with trastuzumab or docetaxel in solid tumor models. Mol Cancer Ther.

[140] Catenacci DVT (2022). MOUNTAINEER-02: Phase 2/3 study of tucatinib, trastuzumab, ramucirumab, and paclitaxel in previously treated HER2+ gastric or gastroesophageal junction adenocarcinoma—Trial in progress. J Clin Oncol.

[141] Wu X, Huang S (2019). HER2-specific chimeric antigen receptor-engineered natural killer cells combined with apatinib for the treatment of gastric cancer. Bull Cancer.

[142] Smyth EC (2020). Gastric cancer. Lancet.

[143] Casak SJ (2015). FDA approval summary: ramucirumab for gastric cancer. Clin Cancer Res.

[144] Fuchs CS (2014). Ramucirumab monotherapy for previously treated advanced gastric or gastro-oesophageal junction adenocarcinoma (REGARD): an international, randomised, multicentre, placebo-controlled, phase 3 trial. Lancet.

[145] Wilke H (2014). Ramucirumab plus paclitaxel versus placebo plus paclitaxel in patients with previously treated advanced gastric or gastro-oesophageal junction adenocarcinoma (RAINBOW): a double-blind, randomised phase 3 trial. Lancet Oncol.

[146] Xu RH (2021). Efficacy and safety of weekly paclitaxel with or without ramucirumab as second-line therapy for the treatment of advanced gastric or gastroesophageal junction adenocarcinoma (RAINBOW-Asia): a randomised, multicentre, double-blind, phase 3 trial. Lancet Gastroenterol Hepatol.

[147] Fuchs CS (2019). Ramucirumab with cisplatin and fluoropyrimidine as first-line therapy in patients with metastatic gastric or junctional adenocarcinoma (RAINFALL): a double-blind, randomised, placebo-controlled, phase 3 trial. Lancet Oncol.

[148] Ohtsu A (2011). Bevacizumab in combination with chemotherapy as first-line therapy in advanced gastric cancer: a randomized, double-blind, placebo-controlled phase III study. J Clin Oncol.

[149] Pavlakis N, et al. INTEGRATE IIa: a randomised, double-blind, phase III study of regorafenib versus placebo in refractory advanced gastro-oesophageal cancer (AGOC)—a study led by the Australasian Gastro-intestinal Trials Group (AGITG). J Clin. Oncol. 2023;41(4):LBA294. 10.1186/s12885-023-10642-7 PMC9945618 36814222

[150] Fukuoka S (2020). Regorafenib plus nivolumab in patients with advanced gastric or colorectal cancer: an open-label, dose-escalation, and dose-expansion phase ib trial (REGONIVO, EPOC1603). J Clin Oncol.

[151] Kawazoe A (2020). Lenvatinib plus pembrolizumab in patients with advanced gastric cancer in the first-line or second-line setting (EPOC1706): an open-label, single-arm, phase 2 trial. Lancet Oncol.

[152] Li J (2016). Randomized, double-blind, placebo-controlled phase III trial of apatinib in patients with chemotherapy-refractory advanced or metastatic adenocarcinoma of the stomach or gastroesophageal junction. J Clin Oncol.

[153] Kang YK (2019). Randomized phase III ANGEL study of rivoceranib (apatinib) + best supportive care (BSC) vs placebo + BSC in patients with advanced/metastatic gastric cancer who failed & 2 prior chemotherapy regimens. Ann Oncol.

[154] Zhang Y, et al. A phase Ib/II study of fruquintinib in combination with paclitaxel as the second-line therapy for advanced gastric cancer. Cancer Commun (Lond), 2022. 10.1002/cac2.12379 PMC9859731 36331272

[155] Sung H, et al. Global cancer statistics 2020: GLOBOCAN estimates of incidence and mortality worldwide for 36 cancers in 185 countries. CA Cancer J Clin. 2021 10.3322/caac.21660 33538338

[156] Sahin U (2008). Claudin-18 splice variant 2 is a pan-cancer target suitable for therapeutic antibody development. Clin Cancer Res.

[157] Hong JY (2020). Claudin 18.2 expression in various tumor types and its role as a potential target in advanced gastric cancer. Transl Cancer Res.

[158] Rohde C (2019). Comparison of Claudin 18.2 expression in primary tumors and lymph node metastases in Japanese patients with gastric adenocarcinoma. Jpn J Clin Oncol.

[159] Sahin U (2021). FAST: a randomised phase II study of zolbetuximab (IMAB362) plus EOX versus EOX alone for first-line treatment of advanced CLDN18.2-positive gastric and gastro-oesophageal adenocarcinoma. Ann Oncol.

[160] Singh P, Toom S, Huang Y (2017). Anti-claudin 18.2 antibody as new targeted therapy for advanced gastric cancer. J Hematol Oncol.

[161] Tureci O (2019). A multicentre, phase IIa study of zolbetuximab as a single agent in patients with recurrent or refractory advanced adenocarcinoma of the stomach or lower oesophagus: the MONO study. Ann Oncol.

[162] Shitara K, et al. Zolbetuximab + mFOLFOX6 as first-line (1L) treatment for patients (pts) withclaudin-18.2+ (CLDN18.2+) / HER2− locally advanced (LA) unresectable or metastatic gastric or gastroesophageal junction (mG/GEJ) adenocarcinoma: primary results from phase 3 SPOTLIGHT study. J Clin Oncol 2023;41(4_suppl): LBA292

[163] Shitara K (2023). Zolbetuximab + CAPOX in 1L claudin-18.2+ (CLDN18.2+)/HER2− locally advanced (LA) or metastatic gastric or gastroesophageal junction (mG/GEJ) adenocarcinoma: Primary phase 3 results from GLOW. J Clin Oncol.

[164] Jiang H (2019). Claudin182-specific chimeric antigen receptor engineered T cells for the treatment of gastric cancer. J Natl Cancer Inst.

[165] Qi C (2022). Claudin18.2-specific CAR T cells in gastrointestinal cancers: phase 1 trial interim results. Nat Med.

[166] Helsten T, Schwaederle M, Kurzrock R (2015). Fibroblast growth factor receptor signaling in hereditary and neoplastic disease: biologic and clinical implications. Cancer Metastasis Rev.

[167] Yue S (2021). FGFR-TKI resistance in cancer: current status and perspectives. J Hematol Oncol.

[168] Bahleda R (2020). Phase I, first-in-human study of futibatinib, a highly selective, irreversible FGFR1-4 inhibitor in patients with advanced solid tumors. Ann Oncol.

[169] Meric-Bernstam F (2022). Futibatinib, an irreversible FGFR1-4 inhibitor, in patients with advanced solid tumors harboring FGF/FGFR aberrations: a phase I dose-expansion study. Cancer Discov.

[170] Lengyel CG, et al. FGFR Pathway Inhibition in Gastric Cancer: The Golden Era of an Old Target? Life (Basel), 2022;12(1) 10.3390/life12010081 PMC8778800 35054474

[171] Catenacci DVT (2020). Phase I escalation and expansion study of bemarituzumab (FPA144) in patients with advanced solid tumors and FGFR2b-selected gastroesophageal adenocarcinoma. J Clin Oncol.

[172] Catenacci DVT (2021). FIGHT: a randomized, double-blind, placebo-controlled, phase II study of bemarituzumab (bema) combined with modified FOLFOX6 in 1L FGFR2b+ advanced gastric/gastroesophageal junction adenocarcinoma (GC). J Clin Oncol.

[173] Nagatsuma AK (2015). Expression profiles of HER2, EGFR, MET and FGFR2 in a large cohort of patients with gastric adenocarcinoma. Gastric Cancer.

[174] Dutton SJ (2014). Gefitinib for oesophageal cancer progressing after chemotherapy (COG): a phase 3, multicentre, double-blind, placebo-controlled randomised trial. Lancet Oncol.

[175] Petty RD (2017). Gefitinib and EGFR gene copy number aberrations in esophageal cancer. J Clin Oncol.

[176] Lordick F (2013). Clinical outcome according to tumor HER2 status and EGFR expression in advanced gastric cancer patients from the EXPAND study. J Clin Oncol.

[177] Maron SB (2018). Targeted therapies for targeted populations: anti-EGFR Treatment for EGFR-amplified gastroesophageal adenocarcinoma. Cancer Discov.

[178] Raj S (2022). Molecular mechanism(s) of regulation(s) of c-MET/HGF signaling in head and neck cancer. Mol Cancer.

[179] Shah MA (2016). A randomized phase II study of FOLFOX with or without the MET inhibitor onartuzumab in advanced adenocarcinoma of the stomach and gastroesophageal junction. Oncologist..

[180] Catenacci DVT (2017). Rilotumumab plus epirubicin, cisplatin, and capecitabine as first-line therapy in advanced MET-positive gastric or gastro-oesophageal junction cancer (RILOMET-1): a randomised, double-blind, placebo-controlled, phase 3 trial. Lancet Oncol.

[181] Doi T, et al. A phase 3, multicenter, randomized, double-blind, placebo-controlled study of rilotumumab in combination with cisplatin and capecitabine (CX) as first-line therapy for Asian patients (pts) with advanced MET-positive gastric or gastroesophageal junction (G/GEJ) adenocarcinoma: the RILOMET-2 trial. J Clin Oncol. 2015;33(3_suppl): TPS226

[182] Kang YK (2014). A phase II trial of a selective c-Met inhibitor tivantinib (ARQ 197) monotherapy as a second- or third-line therapy in the patients with metastatic gastric cancer. Invest New Drugs.

[183] Hong DS (2019). Phase I Study of AMG 337, a highly selective small-molecule MET inhibitor, in patients with advanced solid tumors. Clin Cancer Res.

[184] Shah MA (2013). Phase II study evaluating 2 dosing schedules of oral foretinib (GSK1363089), cMET/VEGFR2 inhibitor, in patients with metastatic gastric cancer. PLoS ONE.

[185] Luo H (2019). Real-time artificial intelligence for detection of upper gastrointestinal cancer by endoscopy: a multicentre, case-control, diagnostic study. Lancet Oncol.

[186] Chen ZH (2021). Artificial intelligence for assisting cancer diagnosis and treatment in the era of precision medicine. Cancer Commun (Lond).

[187] Leal A (2020). White blood cell and cell-free DNA analyses for detection of residual disease in gastric cancer. Nat Commun.

[188] Maron SB (2019). Circulating tumor DNA sequencing analysis of gastroesophageal adenocarcinoma. Clin Cancer Res.

[189] Jin Y (2020). The predicting role of circulating tumor DNA landscape in gastric cancer patients treated with immune checkpoint inhibitors. Mol Cancer.

[190] Wang Y (2022). Immune checkpoint modulators in cancer immunotherapy: recent advances and emerging concepts. J Hematol Oncol.

[191] Upadhaya S (2021). Combinations take centre stage in PD1/PDL1 inhibitor clinical trials. Nat Rev Drug Discov.

[192] Zhao H (2021). Emerging immunological strategies: recent advances and future directions. Front Med.

